# Genetic insights into juvenile idiopathic arthritis derived from deep whole genome sequencing

**DOI:** 10.1038/s41598-017-02966-9

**Published:** 2017-06-01

**Authors:** Laiping Wong, Kaiyu Jiang, Yanmin Chen, James N. Jarvis

**Affiliations:** 1 Department of Pediatrics, University at Buffalo, Buffalo, NY 14203 USA; 2 Genetics, Genomics, & Bioinformatics Program, University at Buffalo, Buffalo, NY 14203 USA

## Abstract

Deep whole genome sequencing (WGS) allows for the comprehensive study of genetic landscapes at finer resolution than array based methods. We conducted deep WGS on children with the polyarticular form of juvenile idiopathic arthritis (JIA), using 2 independent cohorts to ascertain the sequencing fidelity. Genome wide SNP density analysis identified 18 SNP hotspots with comparison to the 1000 Genome Projects (1KGP) data. A subset of the genes adjacent to SNP hotspots showed statistically significant enrichment in immunological processes. Genes adjacent to indel hotspots were functionally related to G-protein coupled signaling pathways. Further analyses elucidated significantly more JIA SNPs with regulatory potential compared to 1KGP data. Furthermore, SNPs located within linkage disequibilium (LD) blocks containing previously identified JIA-associated SNPs demonstrated higher regulation potential compared to SNPs outside LD blocks. We also demonstrated enrichment of novel JIA variants in histone modification peaks and DNase hypersensitivity sites in B cells. This study greatly expands the number of genetic variants that may contribute to JIA and give us some clues into what may trigger this disease. To date, this study is the first deep WGS effort on children with JIA and provides useful genetic resources for research communities particularly in understanding JIA etiology.

## Introduction

JIA is an archetypal complex trait, in which small but measurable genetic susceptibility can be identified in multiple genetic loci, and where the environment contributes significant risk^1^. In JIA, the search for causal variants that contribute to disease risk has been hampered by the small effect sizes^2^. Furthermore, although multiple risk loci for JIA have been identified via candidate gene approaches and genetic fine mapping studies^3, 4^, these approaches carry an inherent bias in their assumption that JIA is an “autoimmune disease”. Genome-wide association studies are free from inherent bias about disease pathogenesis^5^, but these studies provide only a rough estimate of genetic risk loci and are limited in their capacity to detect regions conferring subtle but biologically significant risk. Finally, the task of finding causal variants is further complicated by the fact that known genetic risk for JIA, resides primarily within the non-coding genome^6^.

Whole genome sequencing (WGS), with sufficient depth, may provide, in an unbiased manner, a new opportunity to obtain a finer resolution for regions of genetic risk in complex traits, identify new regions not identified through chip-based methods that query targeted regions on assumptions regarding underlying pathogenic mechanisms. To explore this possibility, we performed WGS on children with polyarticular JIA. We report here multiple new genetic variations and identify epigenetic landscapes surrounding these genetic mutations that may cast light on previously unrecognized disease mechanisms.

## Results

### Reproducibility of genetic variants discovery

As our sequencing data were obtained through two independent sequencing experiments, we conducted a variants concordance check for the SNPs and indels identified in these two sets of data (biological replication). We define concordance rate as a ratio of the number of shared variants between two sets to the total number of variants in the observed set. We observed averages of 77.14% and 66.63% concordance rates for SNPs and indels respectively (Supplementary Figure 1). When we examined genetic variation of a pair of biological replicates from the first batch (technical replication), we saw concordance rates of 99% and 83% for SNPs and indels respectively (Supplementary Table 1). Given these high concordance rates in genetic variant discovery, we determined that we could perform subsequent analyses using the identified genetic variants.

### JIA novel variants discovery

**Figure 1:**
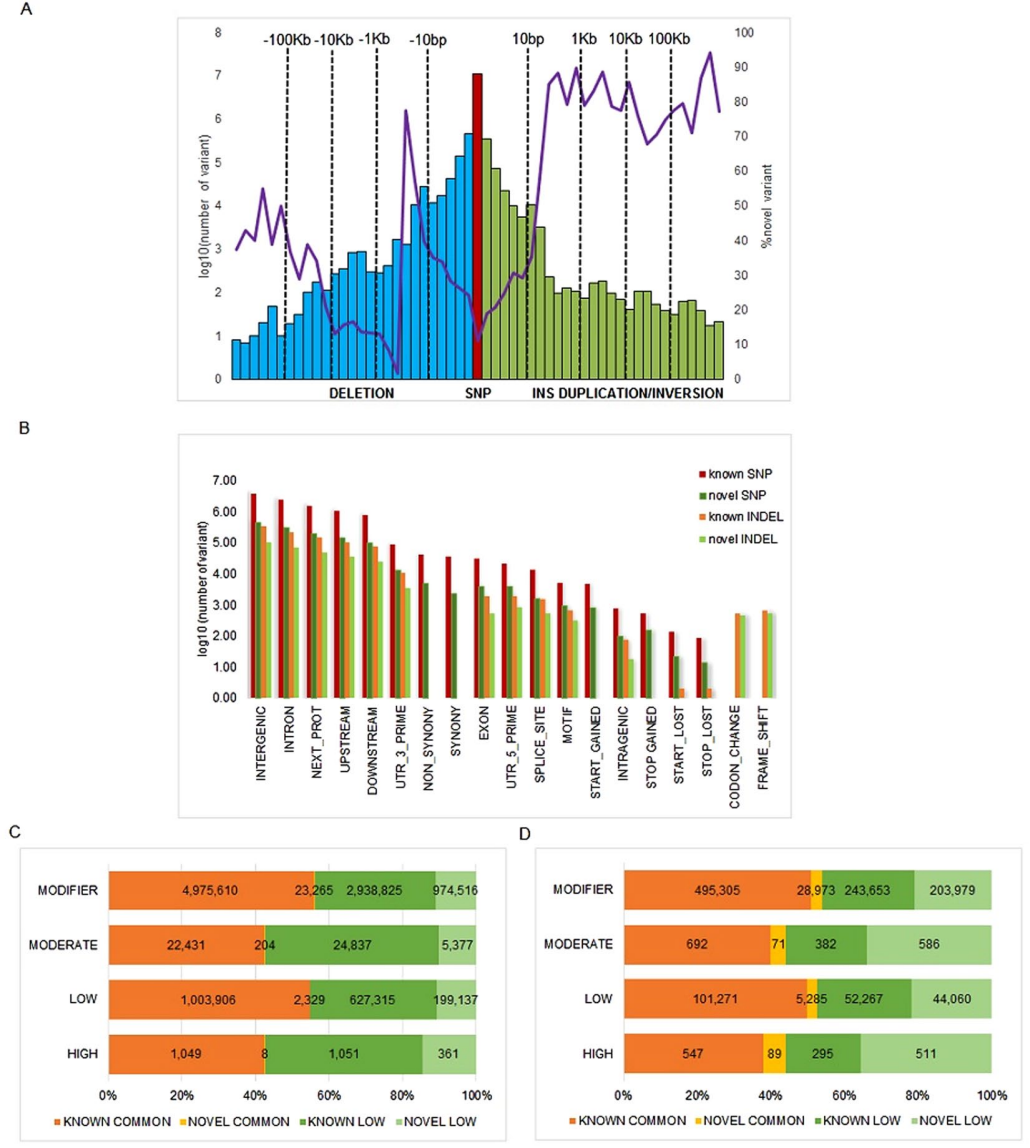
Variants discovered from high coverage DNA whole genome sequencing on 48 JIA individuals. (**A**) Bars show variant numbers (log10) stratified by sizes (horizontal axis) for different type of variant, SNPs are in brown, indels consisting of insertions (green) and deletions (blue). Structural variations (SV, size > 50 bp) in blue represent deletions, green bars are duplications (inversion). The purple line indicates variant novelty rate in percentage. Novelty is defined with respect to dbSNP141 for SNPs and indels whereas novelty for SV is determined by comparing to Database of Genomic Variants archive. (**B**) SNP and indel distribution by genomic features grouped by novelty. Variant distribution based on alternative allele frequency (common >5%, low ≤5%) and variant effects predicted by SNPEff for SNPs (**C**) and indels (**D**).

We discovered a total of 1.49 million novel genetic variants. Specifically, we identified 10,800,221 autosomal bi-allelic single nucleotide polymorphisms (SNPs), of which 6,028,802 (55.88%) are common SNPs, defined as alternative allele frequency AAF >5%. However, 1,205,197 are novel or absent in dbSNP141 (11.2%, Fig. 1A, Table 1, Supplementary Table 2), and 2.14% of these novel SNPs (25,806) are common in all 48 JIA subjects. In addition, we identified 1,177,966 indels (novelty rate of 24%, 632,233 or 53.67% are common indels), 473,261 insertions (96,855 or 20.5% of which are novel) and 704,705 deletions (186,699 or 26.5% of which are novel, Supplementary Table 3). We then defined common structural variants (SVs) as those present in at least 16 out of 48 samples (i.e., present in 33% of the samples). From this analysis, 977 common SV (13.83% of the total novel SVs) were identified. Also, we found multiple novel SV (not reported in DGVa database) within JIA subjects (Supplementary Figure 2). For example, we detected 5,545 deletions (DEL-678 novel), 398 inversions (INV-272 novel) and 1,120 duplications.

**Table 1:** Genetic variants discovered from whole genome sequencing on 48 JIA individuals.

Impact	known		novel	
SNPs	AAF >5%	AAF ≤5%	AAF >5%	AAF ≤5%
HIGH	1,049	1,051	8	361
LOW	1,003,906	627,315	2,329	199,137
MODERATE	22,431	24,837	204	5,377
MODIFIER	4,975,610	2,938,825	23,265	974,516
Indels				
HIGH	547	295	89	511
LOW	101,271	52,267	5,285	44,060
MODERATE	692	382	71	586
MODIFIER	495,305	243,653	28,973	203,979
	**Singleton SNPs**	**Singleton indels**	**LoF SNPs**	**LoF indels**
Average per sample	62,965	6,920	216	236
Maximum	120,411	13,196	233	274
Minimum	48,439	4,207	196	210

Number of JIA SNPs and indels classified by predicted functional impacts and alternative allele frequency (AAF). Lower panel summarizes per sample summary on singleton (private mutation) and loss of function (LoF) variants.

(DUP- 952 novel), resulting in an overall novelty rate of 26.9% (1,902/7,063) for SVs. When we stratified SV by type, we found novelty rates of 12.2%, 68.3% and 85% for DEL, INV and DUP respectively. High novelty rates for INV and DUP were expected, as the DGVa database contained relatively small proportions of INV (1.2%, 1,428) and DUP (17.2%, 21,360) from the total 120,045 SVs in its archive.

Next, we considered Loss of Function (LoF) variants, i.e., variants with potential to disrupt the function of protein coding genes. In total, we found 66,074 LoF variants (62,856 SNPs and 3,218 indels, Supplementary Figure 3, Supplementary Table 4). When we predicted the functional impact of LoF variants, we found 12,517 SNPs (1,924 novel) and 546 indels (320 novel) with potentially detrimental impacts. When we further examined the LoF variants with predicted detrimental effects to seek their regulation potentials, 932 SNPs and 2 indels were annotated with RegulomeDB class 1 regulatory evidence (eQTL, TF binding and DNase peaks, Supplementary Table 5).

We then examined the predicted regulation potential of SNPs discovered from WGS of JIA patients by mapping each SNP’s genomic position to the RegulomeDB database. This procedure resulted in the assignment of SNPs to one of the 13 regulatory evidence classes (Supplementary Table 6). We observed significantly more JIA SNPs grouped under RegulomeDB class 1 (1a–f, the highest number of experimental findings supporting evidence of regulation) compared to 1KGP SNPs (Fisher T-test p-value ≤ 2.2E-16 and 3.8 folds higher in JIA relative to 1KGP, Supplementary Figure 4). Despite the large sample size of 1KGP (n = 2504) versus our cohort (n = 48), we identified more SNPs with class 1 regulation evidence, corroborating the likelihood that these variants are part of the genomic regulatory architecture of the disease process.

In total we identified 40,754 common (alternative allele frequency, AAF ≥5%) and 10,504 low frequency (AAF <5%) JIA SNPs annotated with class 1 regulation evidence. To determine whether those SNPs were genuine mutations or sequencing artifacts, we compared the sequencing depth of 51,258 JIA SNPs with RegulomeDB class 1 regulation evidence (hereafter we denote these SNPs as class 1 JIA SNPs) and all other SNPs (that is, SNPs not annotated with class 1 evidence). This comparison showed that class 1 JIA SNPs exhibited a statistically significant higher sequencing depth than other SNPs (p-value ≤ 2.2e-16). In addition, we examined variant quality and found higher quality scores in class 1 JIA SNPs compared with the other SNPs; higher quality scores indicate higher confidence calls (p-value ≤ 2.2e-16). When we investigated the mapping quality of reads supporting class 1 JIA SNPs and other SNPs, we also identified statistical evidence for better mapping quality for class 1 JIA SNPs compared with the other SNPs (p-value ≤ 2.2e-16). Taken together, the three metrics that quantified the fidelity of the detected SNPs (sequencing depth, variant quality and read mapping quality), make it reasonable to infer that class 1 JIA SNPs are genuine mutation in JIA genomes.

When we performed gene ontology (GO) enrichment analysis using genes of JIA SNPs with class 1 regulation evidence, significant GO terms included carboxylic acid metabolism and ATP hydrolysis coupled proton transport (Supplementary Table 7). These findings are provocative given the wealth of new data emerging regarding the importance of intermediary metabolism in regulating immune function^7^.

### Genome wide distribution of JIA variants

**Figure 2:**
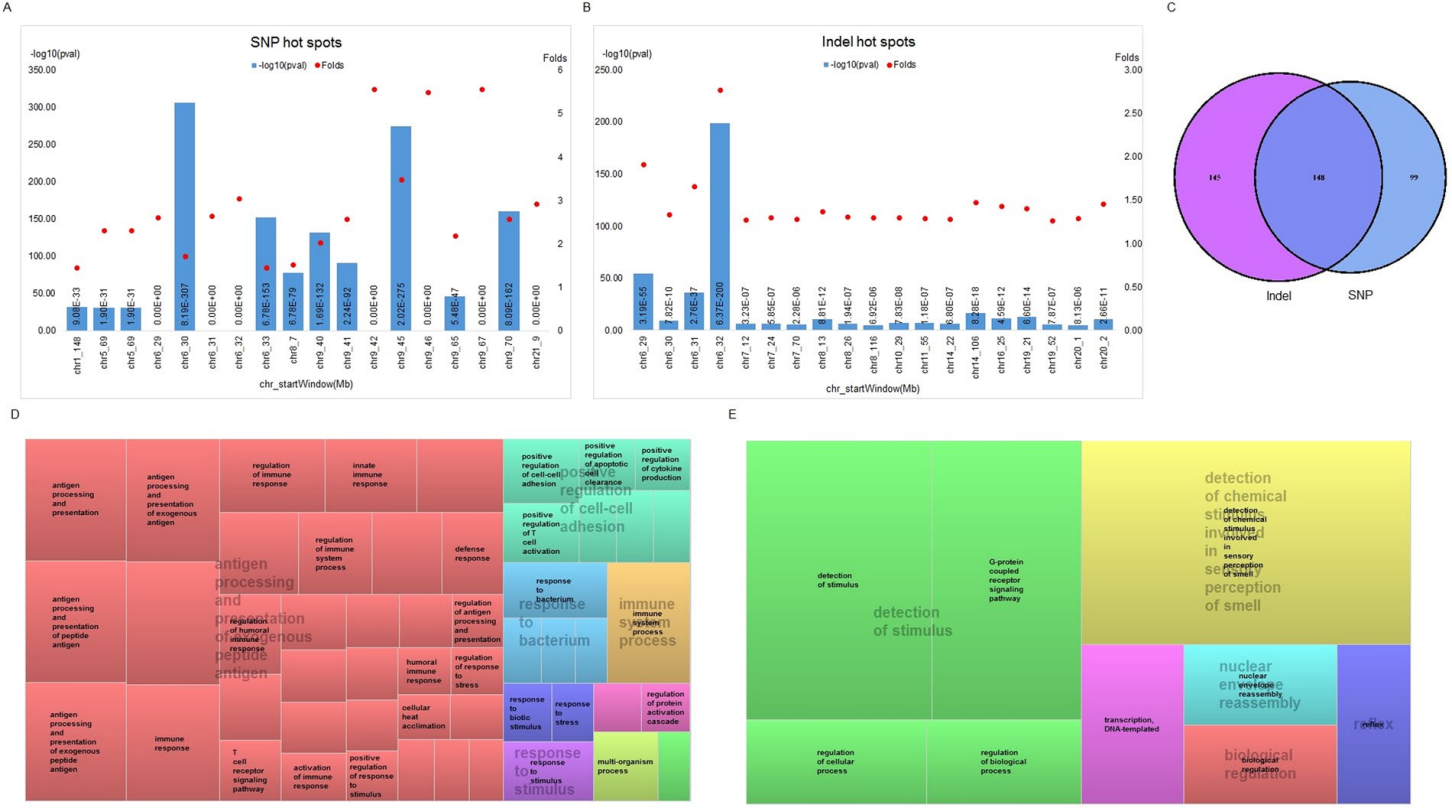
JIA variant hotspots per 1 Mb bin determined with respect to the 1000 Genomes Project variants. The bar shows Fisher exact test p values (-log10) of variant hotspots, and red dots show fold enrichment for each hotspot for (**A**) SNPs and (**B**) indels. (**C**) Venn diagram showing associated genes (overlapped or located within 5 kb upstream and downstream of hotspots) with SNP and indel hotspots. The 247 genes associated with SNP hotspots (of which 148 genes also associated with indel hotspots) were used for Gene Ontology (GO) enrichment analysis. The resulting GO term classifications are depicted using REVigo treemap in (**D**). GO enrichment analysis was also performed with 145 genes from indel hotspots. Results are shown in the GO term clustering represented by REVigo treemap in (**E**). Each rectangle of treemap indicates a GO terms cluster, sub-clusters (related GO terms) of same color are joined into super-clusters (centralized semi-transparent wordings). Size of rectangle reflects statistical significance p-values of GO terms, larger rectangles with smaller p-values.

WGS studies on the JIA samples demonstrated specific genetic variant hotspots within JIA genomes. Genes within these hotspots were annotated to be enriched in immune related biological processes. We defined hotspots as genomic regions (1 Mb bin size) that demonstrated a minimum of 1.5 fold greater density for JIA variants than for the 1KGP variants with Fisher exact test p-values less than 0.05. We identified 18 SNPs hotspots and 19 indel hotspots when comparing JIA WGS with 1KGP data (Fig. 2, Supplementary Table 8). We observed that those variant hotspots are significantly enriched relative to 1KGP with p-value range from 1.9E-31 to 0. Genes associated with SNP hotspots were used as input for biological process GO enrichment analysis using GOrilla software^8^. We identified 46 significant GO biological process terms for genes associated with SNP hotspots (false discovery rate, FDR ≤ 0.05, Supplementary Table 9). REViGO^9^ classified these GO terms into 8 groups. Predictably, we saw enrichment of immune-related biological processes (Fig. 2), compatible with the increasing evidence that suggests that the pathobiology of JIA involves complex interactions between innate and adaptive immunity^10^.

When we annotated genes associated with only indel hotspots, we found 8 significantly enriched GO terms for specific biological processes (FDR ≤ 0.05, Supplementary Table 10). It is interesting to note that 34 genes associated with indel hotspots were enriched in G-protein coupled receptor signaling pathways (Fig. 2). This pathway involves in many pathophysiological processes including pathways related to arthritis^11^, and we have recently demonstrated the importance of genes regulating G-protein coupled receptors in the transcriptional rewiring that characterizes treatment response in JIA^12^.

We next characterized the profiles of SNPs within hotspots and found that novel SNPs have greater regulatory potential compared to known SNPs. In total, we identified 15,994 novel JIA SNPs within the 18 SNP hotspots (2,373 of which were found in at least in 33% of the cohort). Of these, 81 novel SNPs were annotated to have regulation potential (RP) scores ≥0.5, significantly higher than known SNPs (p-value 5.96e-4). When comparing SNPs within hotspots and SNPs outside hotspots, SNPs within hotspots have more regulation potential relative to SNPs outside hotspots (p-value 3.72e-27). A representative genome browser view that depicts regulation properties of JIA variant hotspots is given in Supplementary Figure 5. This figure shows an overlapping of a hotspot region with histone marks (H3K27ac, H3K4me1, H3K27me3 and H3K4me3) found in three immune system related cell types (CD4+ T cells, CD20+ B cells and CD14+ monocytes). This same region has been identified as an expression quantitative trait locus (eQTL) for blood cells in the GTEx database^13^.

### Association of JIA variants with regions of previously identified genetic risk in JIA

We next examined the new variants discovered on WGS in the context of previously identified risk loci for JIA. We found that JIA genetic variants, particularly those SNPs that co-localized within LD blocks containing previously identified JIA-associated SNPs, demonstrated higher regulation potential compared to SNPs outside the JIA risk-associated LD blocks.

The intersection between JIA variants with LD blocks containing regions of known genetic risk for JIA (hereafter denoted as LD blocks), as reported by Hinks *et al*.^3^ and Hersh *et al*.^4^ identified 9,423 SNPs (1,016 novel) and 1,112 indels (261 novel, Supplementary Figure 6). We did not see evidence that the novel JIA variants were specifically enriched within these LD blocks. This was an unexpected result. Although, unlike candidate gene studies and the recently completed genetic fine mapping study^3^, the WGS approach is both unbiased and genome wide, we anticipated seeing new variants at a higher-than-expected frequency in those regions where genetic risk for JIA has already been established. As noted above, however, those SNPs that overlapped the JIA-associated LD blocks showed significant regulatory potential as assessed by the regulatory potential score database. We found significant enrichment of SNPs with RP scores ≥0.5 (p-value 0.0001 and 1.7 fold), indicating that SNPs co-segregating with JIA-associated LD blocks exhibited higher regulation ability compared to SNPs outside those LD blocks.

In order to determine whether the LD blocks were more immunologically active due to JIA genetic mutation or effects of variation from the general healthy cohort, we performed the same analysis on 1KGP data (healthy cohort). We overlapped 1KGP SNPs with LD blocks and obtained SNPs RP scores with reference to UCSC RP score databases. This was followed by generating a contingency table (for Fisher T-test analysis) that contained the number of 1KGP SNPs within (and outside) LD blocks, segregated by RP scores ≥0.5 and less than 0.5. We did not obtain statistical significant evidence of enrichment for the 1KGP SNPs within the LD blocks, i.e., having RP score ≥0.5 relative to those 1KGP SNPs outside LD blocks (p-value 0.6648). This finding supports the hypothesis that those LD blocks are more likely to be immunologically active due to the regulation potential of JIA SNPs, and are not generally impacted by SNPs from healthy subjects (1KGP).

**Figure 3:**
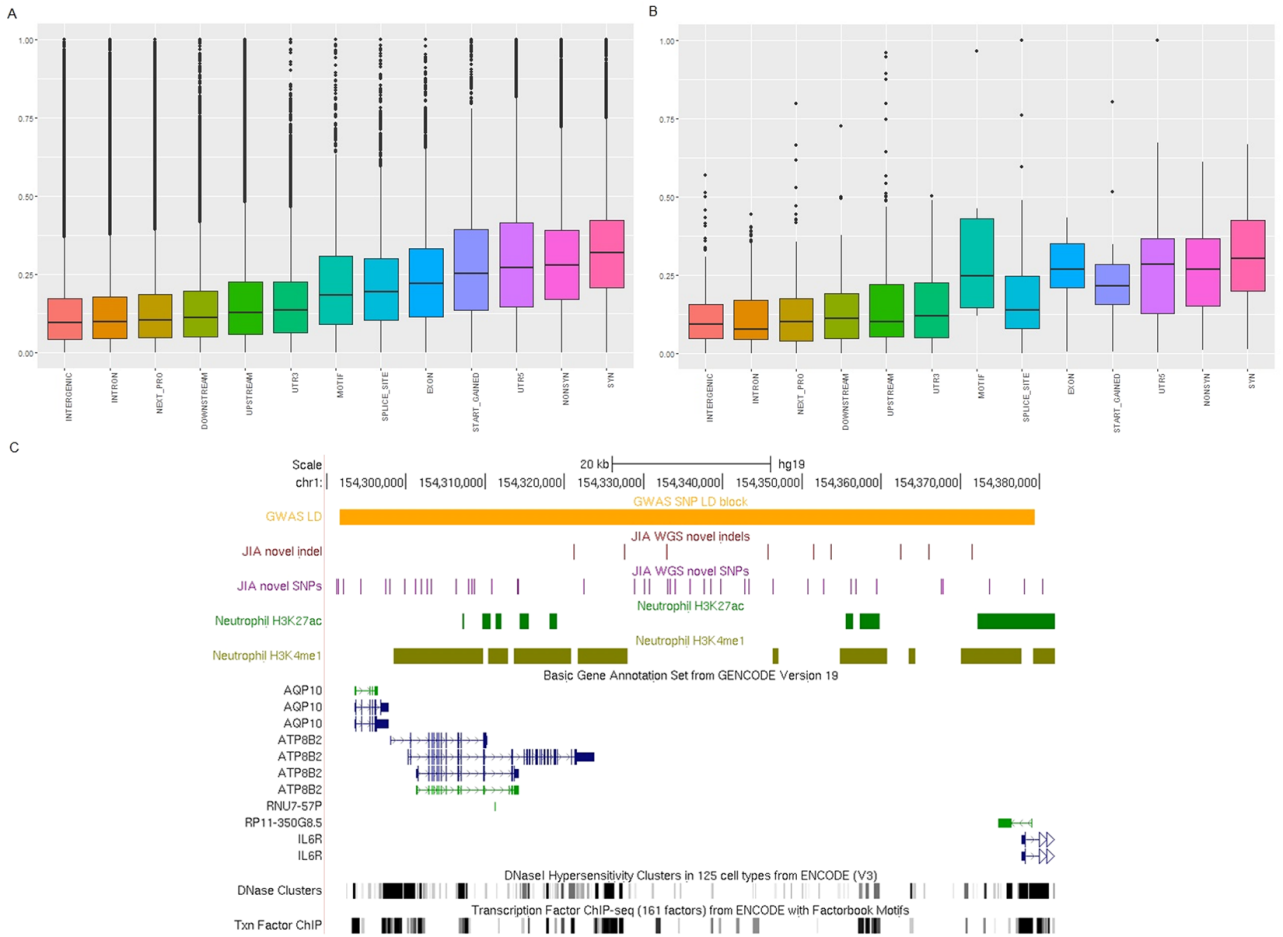
JIA SNPs within linkage disequibilium (LD) blocks containing previously identified JIA-associated SNPs. Boxplot shows regulatory potential scores for SNPs overlapping blocks of JIA-associated SNPs grouped by genomic features for (**A**) all SNPs from WGS on 48 JIA individuals and (**B**) SNPs from WGS on 48 JIA individuals overlapping LD blocks containing previously identified JIA-associated SNPs. (**C**) Genome browser screenshot shows the LD block containing the SNP, rs11265608, intersecting novel JIA SNPs and novel JIA indels. Also, overlapping with neutrophil histone mark H3K27ac and H3K4me1. The gene annotation set from GENCODE v19 is shown by the rows below the H3K4me1 mark. The two rows of black vertical line at the bottom depict DNase hypersensitivity clusters in 125 cell types from ENCODE v3 and the transcription factor ChIP sequencing of 161 factors from ENCODE with factorbook motifs.

In Fig. 3C, we present a representative genomic view of the region adjacent to the LD block containing the JIA-associated SNP, rs11265608^3^, which co-localized with novel SNPs and novel indels from WGS. We note that this region also overlaps with neutrophil H3K27ac and H3K4me1 signals^6^ and spans multiple transcription factor binding sites, suggesting that this is a particularly active genomic region.

In addition to querying the JIA-associated LD blocks, we examined genes (https://ghr.nlm.nih.gov/condition/rheumatoid-arthritis#genes) that are associated with rheumatoid arthritis (RA) and or JIA (Supplementary Table 11, Supplementary Figure 7). We queried for enrichment of novel variants overlapping those associated genes. We did not see statistical significant of enrichment of novel SNPs/indels in arthritis associated gene regions.

### Integration of JIA variants with epigenetic regulatory elements in CD4+ T cells, CD20+ B cells, and CD14+ monocytes

JIA is orchestrated by complex interactions between genetic and epigenetic mechanisms^14^. By leveraging publicly available epigenetic datasets from the ENCODE project^15^ and Roadmap Epigenomics project^16^, we compared JIA genetic variants to histone modification signals, open chromatin accessibility, DNase hypersensitivity sites and CTCF binding sites.

We started with histone modification integration analysis. We observed significant enrichment of JIA novel SNPs within H3K4me3 and H3K27me3 marks in CD20+ B cells (p-value 5.77e-114 and 2.63e-300 respectively). Both of these histone marks identify promoter activities^17^. We also observed that the novel JIA indels were enriched in H3K4me3 peaks of CD4+ T cells (p-value 1.98e-37). We did not observe enrichment of novel JIA variants in H3K27ac or H3K4me1 histone marks in any of the three cell types. In Supplementary Figure 8,, we show a representative genome browser displaying novel JIA variants co-segregating with four histone signals (H3K27ac, H3K27me3, H3K4me1, H3K4me3) annotated in ENCODE and Roadmap Epigenomics data derived from CD20+ B cells, CD4+ T cells, and CD14+ monocytes at the chromosome 6 MHC region.

We next collated JIA SNPs/indels with DNase hypersensitivity sites in all three relevant cells types. DNase hypersensitivity sites mark open chromatin and thus are considered active regions with potential for regulating gene expression^18^. We found that novel JIA SNPs/indels were enriched in the DNase hypersensitivity sites of CD20+ B cells (p-value 3.98e121 and 4.88e-30 for SNPs and indels respectively). In addition, we found that novel JIA SNPs were enriched in CD14+ monocytes DNase hypersensitivity sites (p-value 2.09-e60). We did not see enrichment evidence of novel JIA variants within DNase hypersensitivity sites of CD4+ T cells. We provide an example of a genomic region where novel JIA SNPs/indels overlap DNase hypersensitivity sites of all three immune system cell types in Supplementary Figure 9. At this particular site, the nearest protein coding gene, LRRC37A (leucine rich repeat containing protein 37A), has the primary function of mediating protein-protein interactions and is involved in both innate immunity and nervous system development^19^. This gene is expressed in JIA neutrophils^6^ (maroon and light green rows of Supplementary Figure 9); note the presence of DNase hypersensitivity signals at adjacent loci (dark green peak signals).

Finally, we intersected JIA SNPs/indels with CTCF binding sites of three relevant cell types as a method for screening for potential underlying alterations in transcription factor binding sites due to genetic variation. This analysis yielded no statistical evidence of novel JIA variants enrichment within CTCF binding sites.

## Discussion

The role of genetic risk for JIA has been well-established by family studies^20^ as well as the higher than expected prevalence of other autoimmune or chronic inflammatory diseases in the families of affected children^21^. However, our knowledge of the genetic landscape for this family of illnesses remains incomplete. Furthermore, the two largest GWAS^22, 23^ combined patients with 2 phenotypically different subtypes (oligoarthritis and polyarthritis), so subtype-specific data are still lacking.

Deep WGS provides an unprecedented opportunity to comprehensively study genetic landscapes at finer resolution than can be achieved with chip-based methods. While previous candidate gene approaches and genome-wide association studies have revealed useful information about genetic risk in JIA, a finer mapping is needed to decipher genetic landscapes of children with this disease to gain insights into pathogenesis. For example, even with dense genotyping using the Illumina Immunochip, Hinks *et al*.^3^ were able to explain no more than 18% of the risk for JIA. We therefore conducted deep WGS on 48 children with the polyarticular form of JIA over 2 independent cohorts, comparing results with publically available WGS data from healthy individuals (1KGP). The JIA specific genetic variation achieved an average of 72% concordance between the 2 cohorts despite the ethnic heterogeneity between them.

Using WGS, we were able to identify multiple new loci within JIA genomes that can now be considered candidate JIA risk regions suitable for further investigation. These loci consist of “hotspots” that were enriched for genetic variants discovered by WGS. Chromosome 6, where the major histocompatibility (MHC) genes are located, was a particularly prominent hotspot, supporting the existing view that JIA pathogenesis involves aberrant adaptive immune responses. However, the MHC locus is extraordinarily complex, and, in addition to genes that regulate the T cell responses, is rich in non-coding functional elements that include H3K4me1/H3K27ac-marked enhancers and non-coding RNA species in several relevant cell types^24^. Thus, the presence of risk-associated variants within this genetically-rich region should not be taken as *prima facie* evidence that the causal variants include or are limited to MHC molecules. In the final analysis, functional studies in a broad spectrum of relevant cells will be required to identify causal variants. While this work has significantly expanded the variants that need to be queried, technologies now are available to accomplish this task^25^.

This work corroborates the importance of the non-coding, functional genome in conferring risk for JIA. We have recently reported that the loci reported by Hinks *et al*.^3^ are enriched for H3K4me1/H3K27ac-marked enhancers in both CD4+ T cells and neutrophils^6^. In the current study, the newly-identified genetic variants were located within regions of functional elements in CD4+ T cells and CD20+ B cells as annotated by the ENCODE and Roadmap Epigenomics projects. Thus, JIA resembles almost every other complex trait, in which genetic risk lies largely in the functional, non-coding genome. This means that the field will be faced with the task of performing functional studies in a broad spectrum of cells, as there is no way to determine, a priori, the specific cell type in which genetic risk is most likely to operate. The appearance of JIA-associated genetic variants within functional elements in B cells, for example, was unexpected. Our findings invite additional inquiry into the role of B cells in this disease.

One finding of this JIA genomics study is that it corroborates the importance of previously identified JIA genetic risk loci. When we examined the newly identified genetic variants, those located within the LD blocks containing the known risk-associated SNPs had higher regulatory potential than variants situated outside those LD blocks. On the other hand, when using 1KGP SNPs, we did not observed higher regulation potential within those LD blocks than 1KGP SNPs outside LD blocks, indicating that JIA SNPs within LD blocks very have functional significance. As noted above, however, our current work suggests that functional studies will need to be performed on a broad spectrum of cell types in order to clarify the causal link between genetic variance and immune dysfunction in JIA.

To date, this is the first deep WGS on JIA samples, serve as important data resource that has been instrumental in driving the progress biomedical and clinical research. We envision such data resource can often be benchmarked to deliver scientific impact in such a way that it facilitates the design of future hypothesis or experiment. Furthermore, genomic information from this data resource could lead to translational impact typically guides the changing of clinical practices, imposing clinical validity and industry relevance.

We are aware that these studies are just scratching the surface of where we want to go in building multi-dimensional genomic models for JIA. Such models will invariably include *disease-specific* epigenetic data, and concomitant genetic studies to determine whether and how underlying genetic variation alters epigenetic marks and genome function in pathologically-relevant cells. We are now learning that disease states are characterized by specific epigenetic signatures^26, 27^. Recently, Peeters *et al*. identified^28^ novel enhancer marks in memory T cells derived from synovial fluid of children with JIA. The degree to which these regulatory elements are essential to the disease process or even JIA-specific remains uncertain, however. Enhancer localization in inflammatory cells is highly dependent on the local microenvironment^29, 30^, and it seems likely that similar findings would emerge from the study of inflammatory cells located at any site of chronic inflammation.

In summary, these studies demonstrate the utility of WGS, even on relatively small sample numbers, for elucidating underlying genetics of JIA. Segregation of variants from WGS of JIA individuals within known disease-associated LD blocks overcomes the limitation of depth and coverage of arrays in searching for JIA risk variants. Distribution of JIA SNPs/indels in regulatory elements serves as valuable resource from which to develop insights into epigenetic alterations underlying the effects of genetic variants. The long-term goal for this genomic study will be to integrate existing (and newly-developed) epigenetics resources and prospective JIA clinical records. Based on such data, “Precision Medicine” clinical decisions can be efficiently made to address clinical needs as well as answering important questions about JIA disease pathogenesis, clinical course, and the underlying biology of treatment response.

## Materials and Methods

### Samples

We performed WGS using Illumina X Ten on 50 samples from 48 individuals with JIA over two independent cohorts (Supplementary Table 12, 37 girls and 11 boys). All children fit International League Against Rheumatism criteria for rheumatoid factor negative, polyarticular JIA^31^. Fourteen of these children had detectable antinuclear antibodies at presentation. There were 29 samples in the first batch (B1), which included one pair of technical replicates (one subject was sequenced twice), while the second batch (B2) consisted of 19 subjects. All samples came from children of European or mixed European-American Indian ancestry and were obtained via a University of Oklahoma Institutional Review Board approved protocol. Informed consent was obtained from the parents of all patients. All research was carried out in accordance with the IRB-approved protocol and in compliance with relevant state and Federal regulations.

### Sequencing data

Over all 48 samples, we obtained 39.81 billion properly paired-end reads (2 × 150 bp), aligned using Burrows-Wheeler Aligner (BWA)^32^ to the human reference genome GRCh37. An average of 97.87% reads per sample were mapped to the human reference genome (Supplementary Figure 10). A per sample average insert size of 352 bp was observed, meeting the targeted sequencing insert sizes of 300–400 bp (Supplementary Table 13). Furthermore, a mean sequencing depth at 38X across 48 samples, fulfilled the targeted 30X coverage (Supplementary Figure 11).

### Variant discovery

We adopted the Genome Analysis Toolkit (GATK)^33, 34^ practices in calling SNPs and small insertions or deletions (indels, 1–50 bp). In order to identify high quality variants, we combined GATK variant quality score recalibration filtering and retained SNPs/indels that passed filtration criteria consisting of read depth ≥20X, genotype quality ≥20, variant quality ≥30 and minor-read ratio (MRR) ≥0.2. To access variant novelty, we denoted novel variants (SNPs/indels) as those that were absent in dbSNP141 build 37^35^ and known variants as those present in the dbSNP141.

For structural variation (SV, 100–100 M bp), we used DELLY^36^ to detect deletions (DEL), duplications (DUP), and inversions (INV), with reads of mapping quality ≥20. We denoted novel structural variants (SVs) as those absent in Database of Genomic Variants archive (DGVa)^37^.

### WGS quality control

We first performed sample ethnicity screening by applying principal component analysis (PCA) over genotype information of JIA SNPs and 1KGP SNPs to compare our sample ethnicities against the reference to 1KGP data. This resulted in the JIA samples clustering together with the American samples of 1KGP (Supplementary Figure 12), as would be expected from the population distribution of the studied subjects.

We next examined per sample variant heterozygous to homozygous ratios (Het/Hom) to ensure that the ratio fell within the expected range of genomic scale at 1.5.^38^ We computed an average of 1.6 Het/Hom per sample (Supplementary Table 14). We also examined transition to transversion ratios (Ti/Tv) for each sample, resulting in an average of 2.11 Ti/Tv per sample (Supplementary Table 15; the expected Ti/Tv is around 2.0^39^).

Finally, for variant discovery QC, we examined the distribution of genetic variants across all autosomal chromosomes for each sample. We found that all samples demonstrate similar numbers of discovered variants (SNPs, indels and SV) at the same chromosomes (Supplementary Figure 13).

### Variant annotation

SNPEff^40^ was used for functional annotation of bi-allelic SNPs and indels. An in-house customized script was used to summarize the annotation results stratified by functional impacts (HIGH, MODERATE, LOW and MODIFIER), novelty, alternative allele frequency and genomic features (Supplementary Table 16).

In order to assess the potential functional significance of JIA SNPs, we mapped JIA SNPs to the regulatory potential (RP) scores^41^ downloaded from UCSC repository. The RP score measures similarity of patterns in alignments to those in known regulatory regions^42^. RP score ranges between 0 and 1, high score indicates high regulation potential.

Next, we annotated loss of function (LoF) variants following the definition used by MacArthur *et al*.^43^. LoF SNPs were categorized to one of the following genomic features: non-synonymous, start lost, stop gained, stop lost and splice site. Indels that are classified as frameshift or splice sites are considered LoF indels. We predicted the functional effects of LoF SNPs using Polyphen^44^ and categorized the prediction into two classes (damaging and benign). For LoF indels, we used the SIFT INDEL^45^ bioinformatics tool to predict indels’ functional effects as either damaging or benign. In addition, we conducted regulation potential predictions for LoF variants by mapping them to the RegulomeDB^46^ database to obtain the associated regulation evidence (Supplementary Table 6).

### Variant distribution

To investigate the distribution of SNPs and indels within JIA genomes, we computed variant density by counting number of variants in non-overlapping bins of 1 M bp size over the entire genome. To identify variant hotspots, we compared JIA SNPs (indels) density and 1KGP SNPs (indels) density, i^th^ bin with the fold ratio given by:

$${{\rm{fold}}}_{{\rm{i}}}=\frac{{\rm{num}}\_{\rm{JIA}}\_{\rm{Var}}\_{{\rm{bin}}}_{{\rm{i}}}/{\rm{total}}\_{\rm{JIA}}\_{\rm{var}}}{{\rm{num}}\_1{\rm{KGP}}\_{\rm{Var}}\_{{\rm{bin}}}_{{\rm{i}}}/{\rm{total}}\_1{\rm{KGP}}\_{\rm{var}}},$$

where num_JIA_Var_bin_i_ is the number of JIA variant in i^th^ bin and total_JIA_var represents genome wide total number of JIA variants. Similarly, num_1KGP_Var_bin_i_ is the number of 1KGP variant in i^th^ bin and total_1KGP_var represents genome wide total number of 1KGP variants, noting that 1KGP samples as healthy controls for this variant distribution analysis. We used a ratio of at least 1.5 and a Fisher exact test p-value ≤ 0.05 to define SNP/indel hotspots. For the detected variant hotspots, we searched for associated genes, arbitrarily designated as those genes located within the hotspot regions including genes located 5kbp upstream and downstream of hotspot regions. We then conducted gene ontology (GO) enrichment analysis using Gene Ontology enRIchment anaLysis and visuaLizAtion tool (GOrilla)^8^ to assess the functions of the protein encoding genes within the variant hotspots. REduce Visualize gene ontology (REVigo) was used to classify GO terms base on semantic similarity measurement^9^.

### Variant association with regions of previously identified genetic risk for JIA

We examined JIA SNPs/indels that are located within regions of JIA genetic risk based on the previously identified LD blocks containing JIA-associated SNPs from studies by Hersh *et al*. and Hinks *et al*.^3, 4^. Bedtools^47^ was used to intersect the discovered JIA SNPs/indels within those LD blocks (Supplementary Table 17). We then performed novel variant enrichment analysis using Fisher exact test. LD block information was obtained using the SNAP database^48^ with reference to the 1KGP pilot1 at cutoff of r^2^ > 0.8 and distance limit of 500 kilobases.

In addition, we collected a list of genes reported to have an association with arthritis from literature reviews (Supplementary Table 11) and intersected those genes with JIA SNPs/indels using bedtools.

### Variant association with epigenetic regulatory elements of CD4+ T cells, CD20+ B cells and CD14+ monocytes

Interplays between epigenetic mechanisms and genetic variants may reveal important phenotype genotype relationships^49^. Therefore, we chose pathologically relevant cell types, i.e., CD4+ T cells^50^, CD20+ B cells^51^ and CD14+ monocytes^52^, to elucidate possible associations of JIA SNPs/indels and epigenetic elements (histone modifications, open chromatin and CTCF binding sites). We focused on four histone marks: H3K27ac, H3K4me1, H3K27me3 and H3K4me3, for integration with JIA genetic variants. For this analysis, we downloaded histone modification ChIP sequencing (ChIPSeq) data from GEO databases (Supplementary Table 18). Raw ChIPSeq reads were mapped to the human reference genome GRCh37 using BWA. Mapped reads were then used for peak calling by Model based Analysis of Chip-Seq (MACS2)^53^, with default parameter settings for each histone mark independently. Next, we searched for co-localization between JIA SNPs/indels and chromatin accessible genomic regions. We obtained DNase hypersensitivity site data from the ENCODE project. We also investigated the co-segregation of JIA genetic variants and CTCF binding sites, which regulate gene expression through the organization of three dimensional chromatin structure^54^. For CD4+ T cells we used CTCF binding site data identified by Cuddapah *et al*.^55^ whereas for CD20+ B cells and CD14+ monocytes, we obtained the data from ENCODE (Supplementary Table 18).

We used bedtools to intersect the aforementioned epigenetic elements and JIA SNPs/indels. Enrichment analysis of novel SNPs/indels was conducted by Fisher exact test relative to known variants.

## Electronic supplementary material

Supplementary Information
