# Supplementary material for: Genetic insights into juvenile idiopathic arthritis derived from deep whole genome sequencing
Source: Sci Rep. 2017 Jun 1;7:2657. doi: 10.1038/s41598-017-02966-9 (PMC5453970; doi:10.1038/s41598-017-02966-9)
Supplement: Supplementary file 1 — Supplementary Information [file 41598_2017_2966_MOESM1_ESM.pdf]

Supplementary Information

Genetic insights into juvenile idiopathic arthritis derived from deep whole genome sequencing

Laiping Wong[1], Kaiyu Jiang[1], Yanmin Chen[1], James N. Jarvis[1,2*]

[1]Department of Pediatrics, University at Buffalo, Buffalo, NY

[2]Genetics, Genomics, & Bioinformatics Program, University at Buffalo, Buffalo, NY

Email addresses

Laiping Wong: laiping.wong@roswellpark.org

Kaiyu Jiang: kaiyujia@buffalo.edu

Yanmin Chen: yanminch@buffalo.edu

James N. Jarvis: jamesjar@buffalo.edu

Supplementary Figures

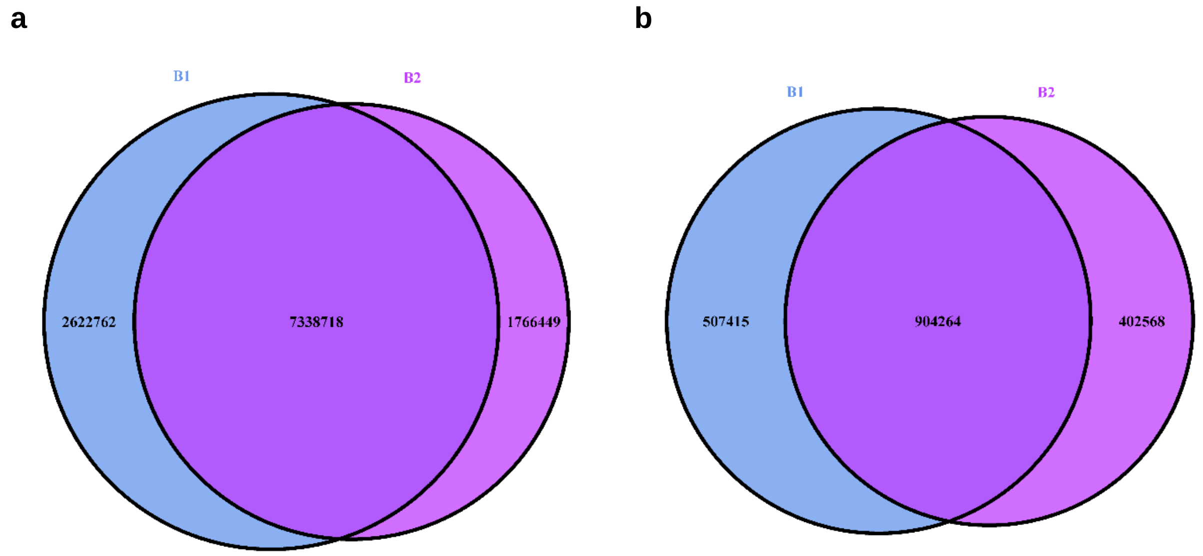

Supplementary Figure 1. Venn diagram showing variants discovered from 2 batches (first batch B1, second batch B2) of whole genome DNA sequencing on JIA patients for (a) SNPs, average 77.14% concordance rate [(7338718/(7338718+2622762)+ 7338718/(7338718+1766449))/2*100] and (b) indels, average 66.63% concordance rate [(904264/(904264+507415) + 904264/(904264+402568))/2*100].

a

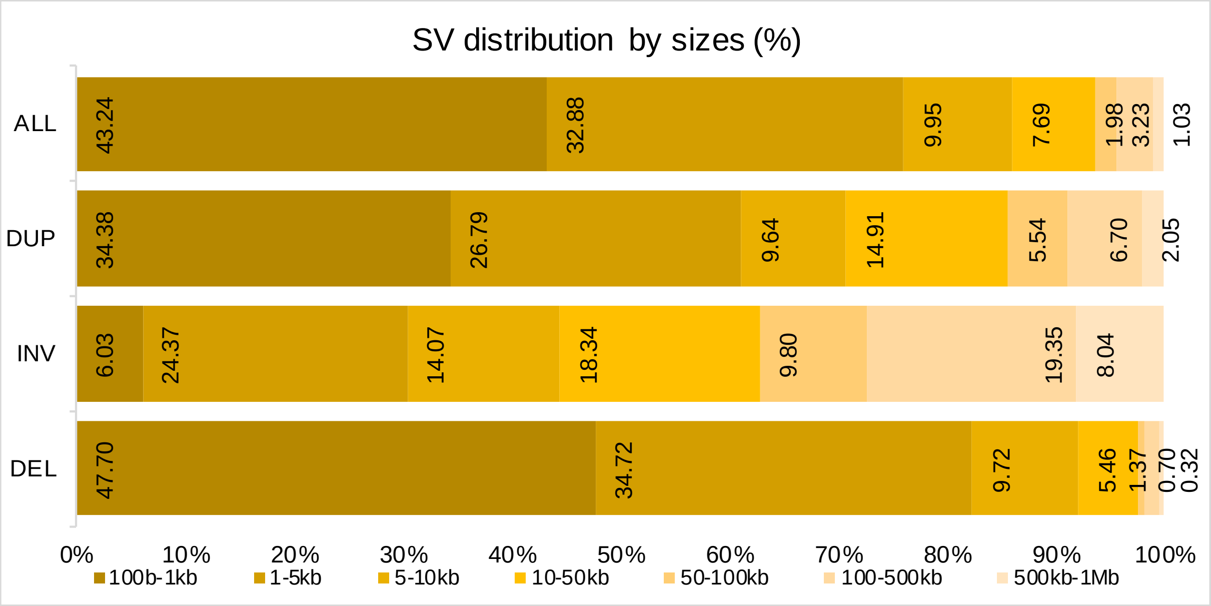

b

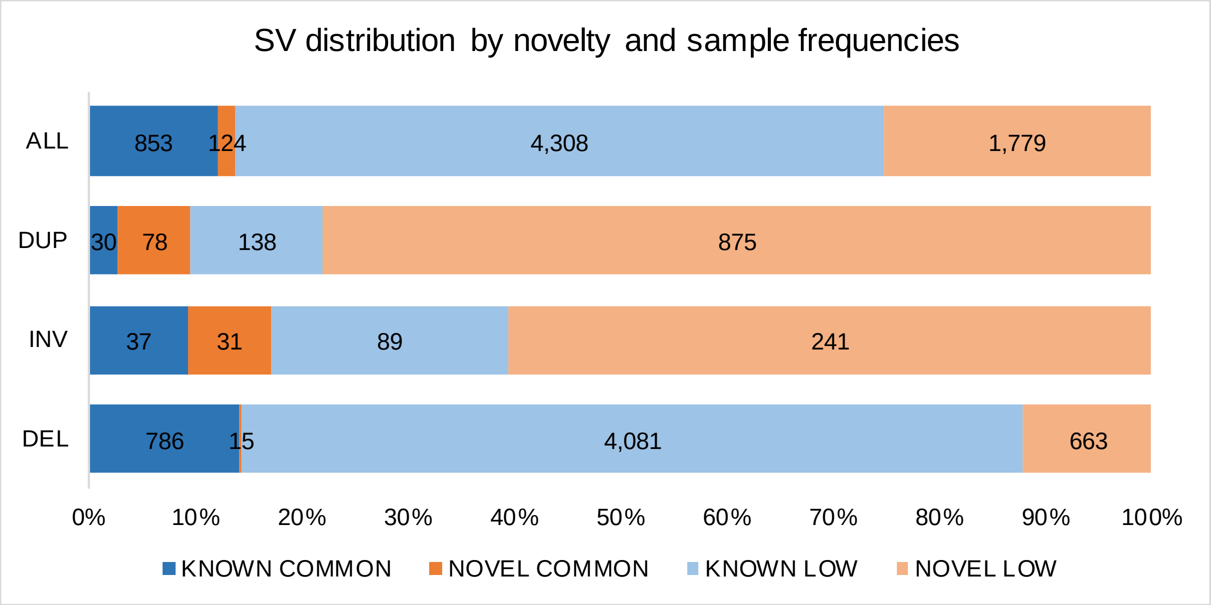

Supplementary Figure 2. Structural variation (SV) distribution by size. Structural variation (SV) discovered from whole genome sequencing on JIA patients. (a) The bar shows the distribution of SV over different size ranges between 100bp-1Mbp. The number inside each sub-block within the bar is the percentage of SV of the indicated size. DEL: deletion, INV: inversion, DUP: duplication. (b) The bar plot displays SV distribution by novelty and sample frequencies. Novel SVs are variations not present in Database of Genomic Variants. The largest number of SV fall within 100bp-1kbp in size with smaller numbers of SVs being 500kbp-1Mbp in size. Also, 14% SVs are common in the cohort.

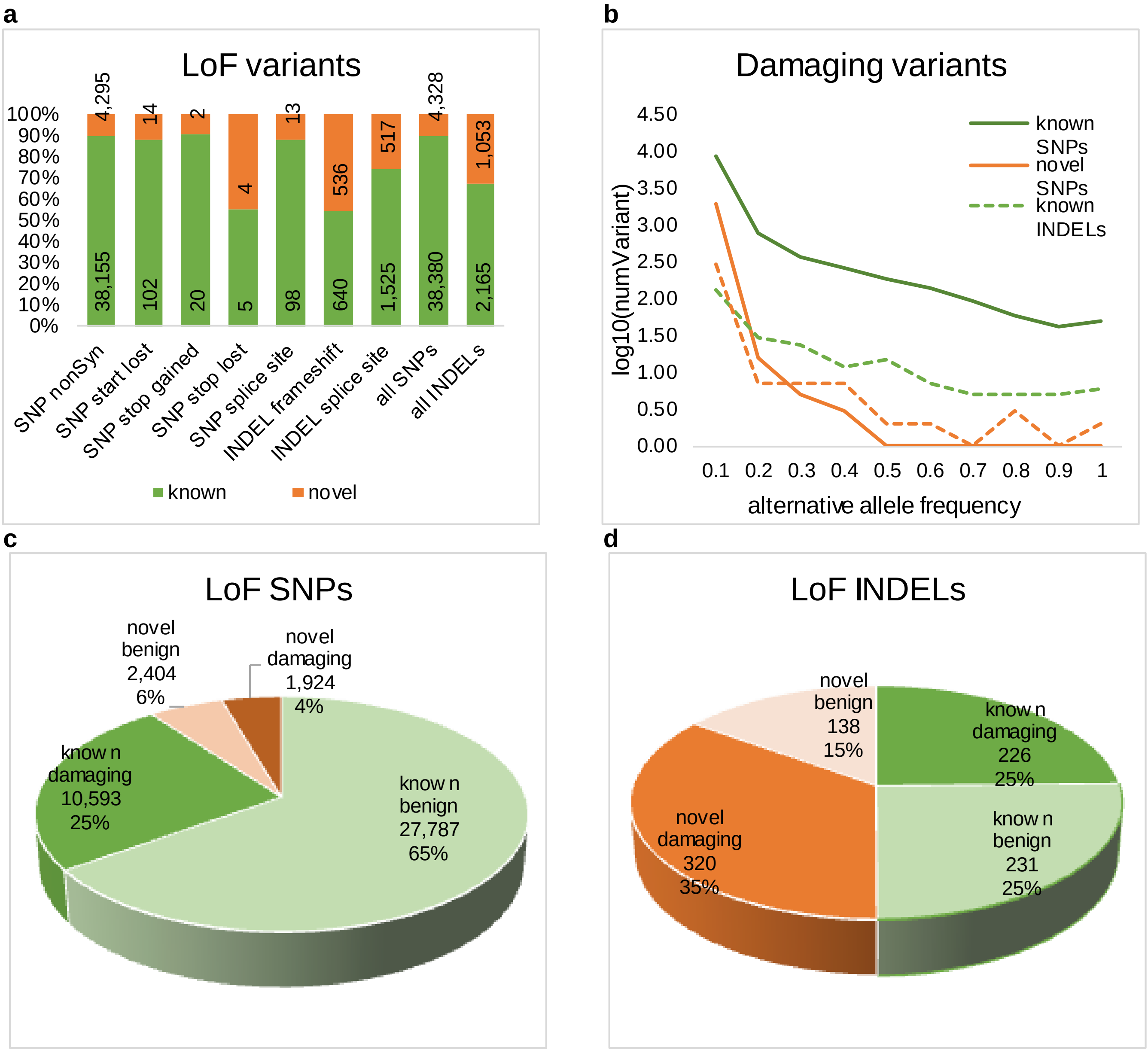

Supplementary Figure 3. Loss of function (LoF) variants discovered from whole genome DNA sequencing on 48 JIA patients. (a) LoF variants distribution based on novelty and genomic features. (b) Distribution of LoF variants with predicted damaging effects by alternative allele frequency. Distribution of LoF (**C**) SNPs and (**D**) indels categorized by predicted functional effects (benign or damaging) and novelty.

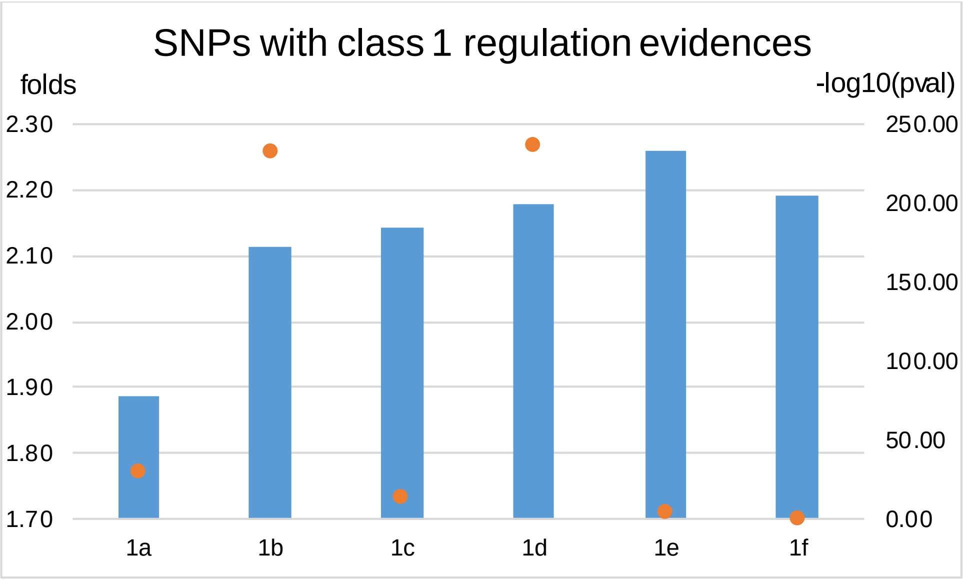

Supplementary Figure 4. SNPs with Class 1 regulation evidence. Enrichment analysis of JIA SNPs with class 1 regulation evidences based on RegulomeDB relative to 1000 Genome Projects SNPs. The bar indicates enrichment fold (left vertical axis), and orange dot represents Fisher exact test p-values in -log10 format (right vertical axis).

a

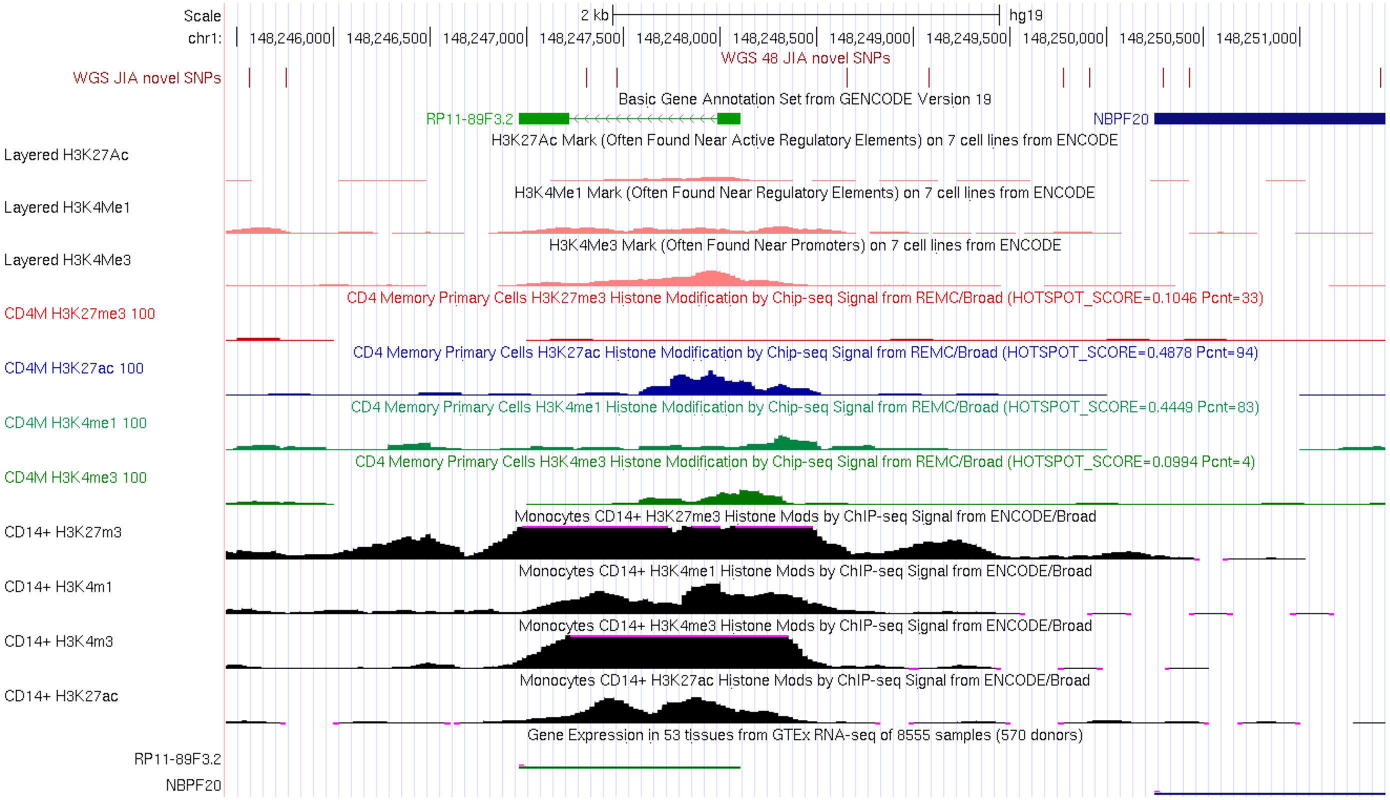

b

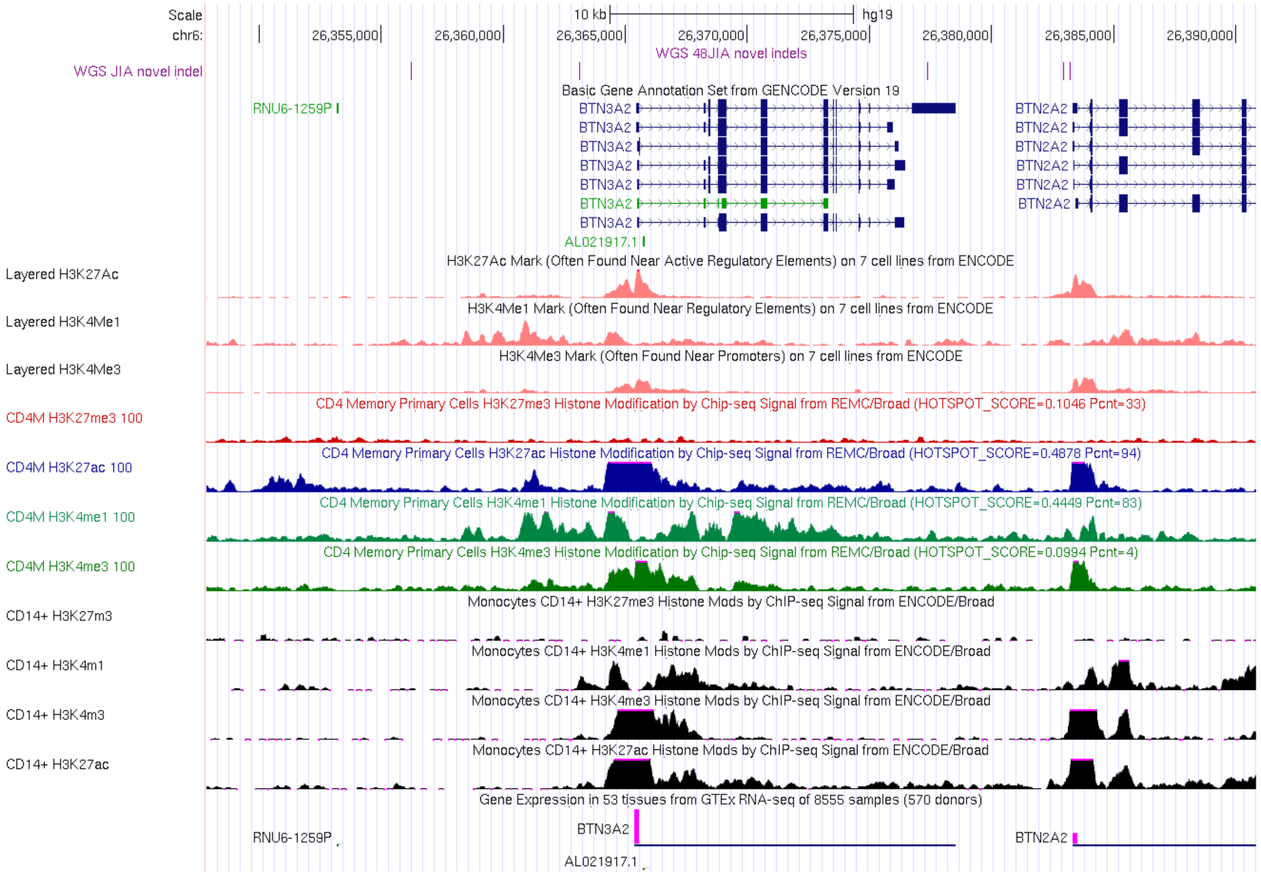

Supplementary Figure 5. Regulation properties of JIA variant hotspot. Screenshot from the UCSC genome browser showing novel JIA (a) SNPs and (a) indels overlapping with histone marks (H3K27ac, H3K27me3, H3K4me1, H3K4me3) from CD20+ B cells, CD4+ T cells, and CD14+ monocytes as well as expression quantitative trait loci (eQTL) signals from the GTEx database, focused on blood tissue data (the last row).

a

b

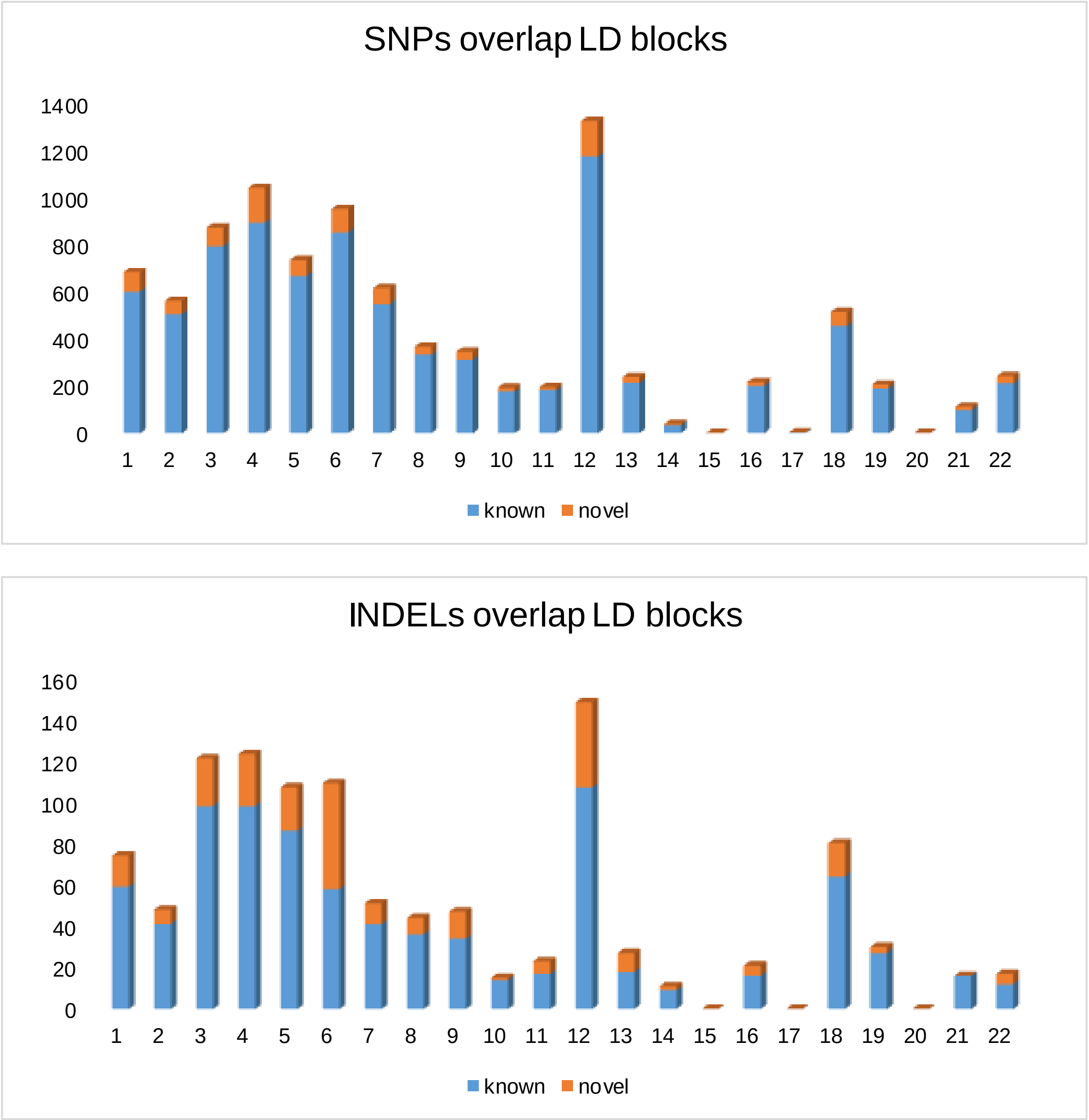

Supplementary Figure 6. SNPs/indels overlapping LD blocks. Intersection of genetic variants from whole genome DNA sequencing on JIA patients with previously identified linkage disequibilium (LD) blocks containing JIA associated SNPs. Bars represent the number of overlapping known (blue) or novel (orange) variants for (a) SNPs and (a) indels on each chromosome (horizontal axis).

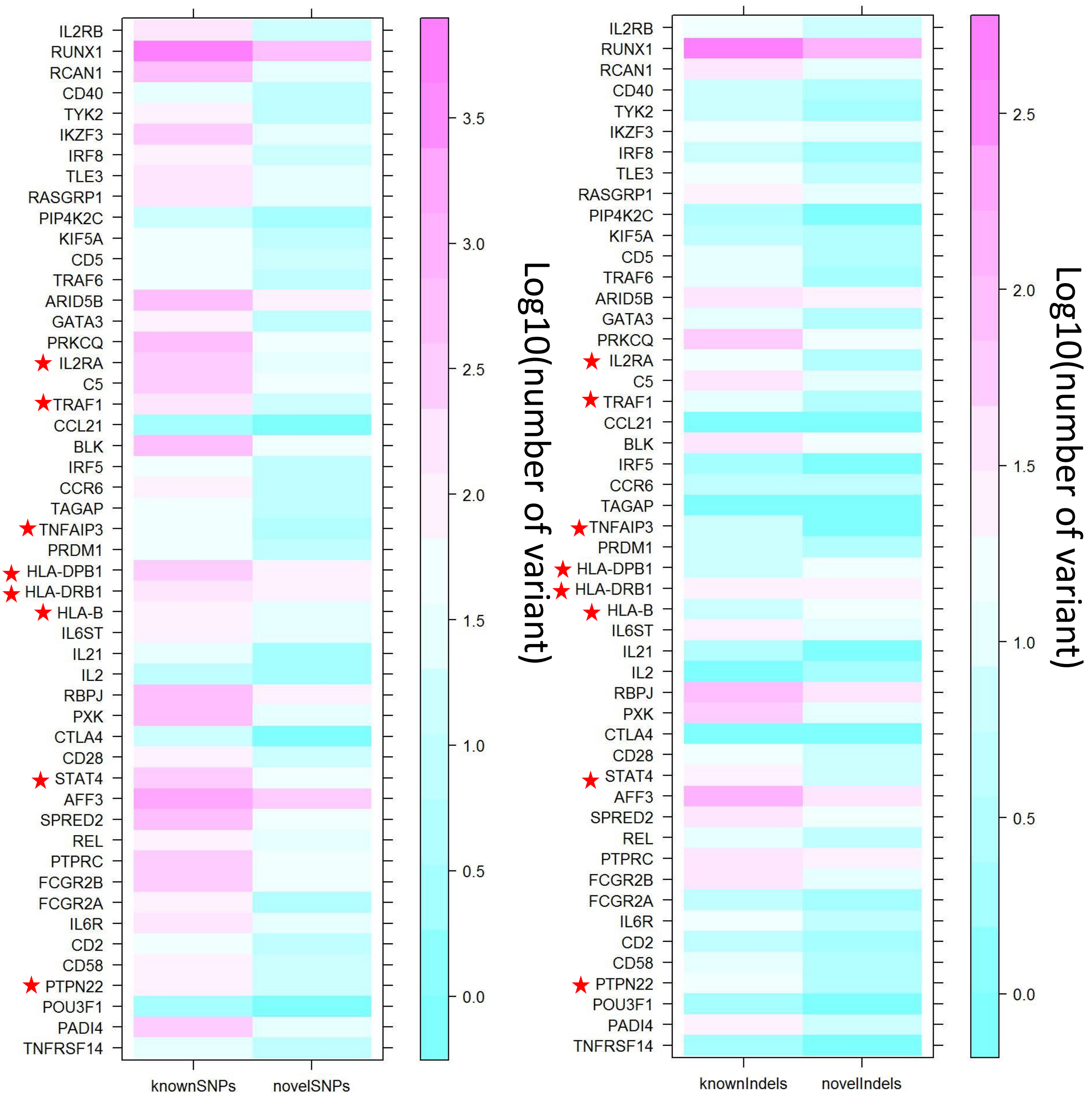

Supplementary Figure 7. Heatmap of variants from WGS on JIA patients co-localized with genes previously shown to be associated with arthritis. Each row represents a gene and each column indicates a known or novel variant .Genes associated specifically with JIA are marked with a red star. The left panel shows data for SNPs and right panel shows data for indels.

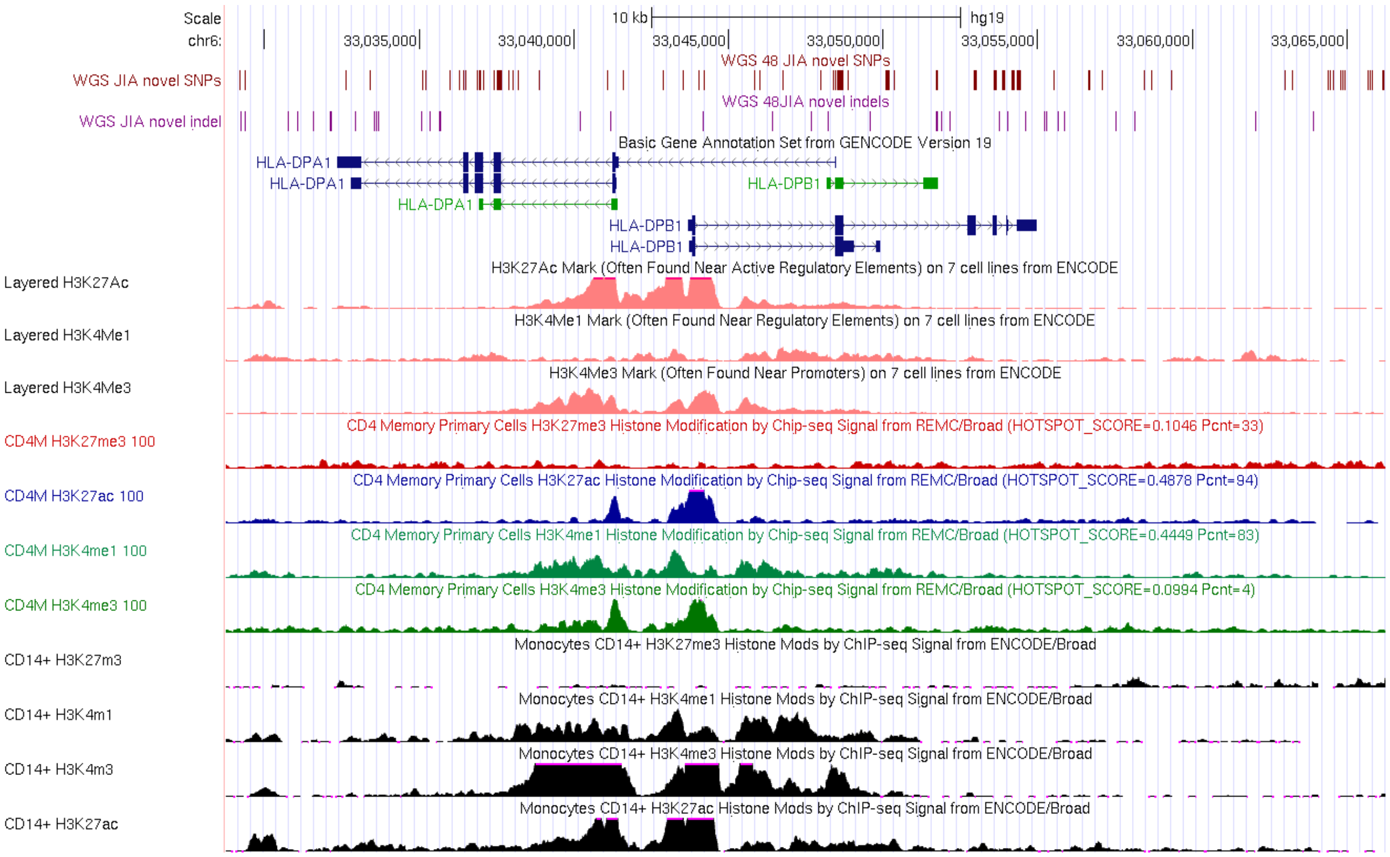

Supplementary Figure 8. Co-segregation of JIA novel SNPs/indels with histone modification marks. Genome browser view showing adjacent regions of HLA-DPB1 gene on chromosome 6 containing novel JIA SNPs/indels. This region also shows abundant histone signals (H3K27ac, H3K27me3, H3K4me1, H3K4me3) from CD20+ B cells (layered), CD4+ T cells, and CD14+ monocytes.

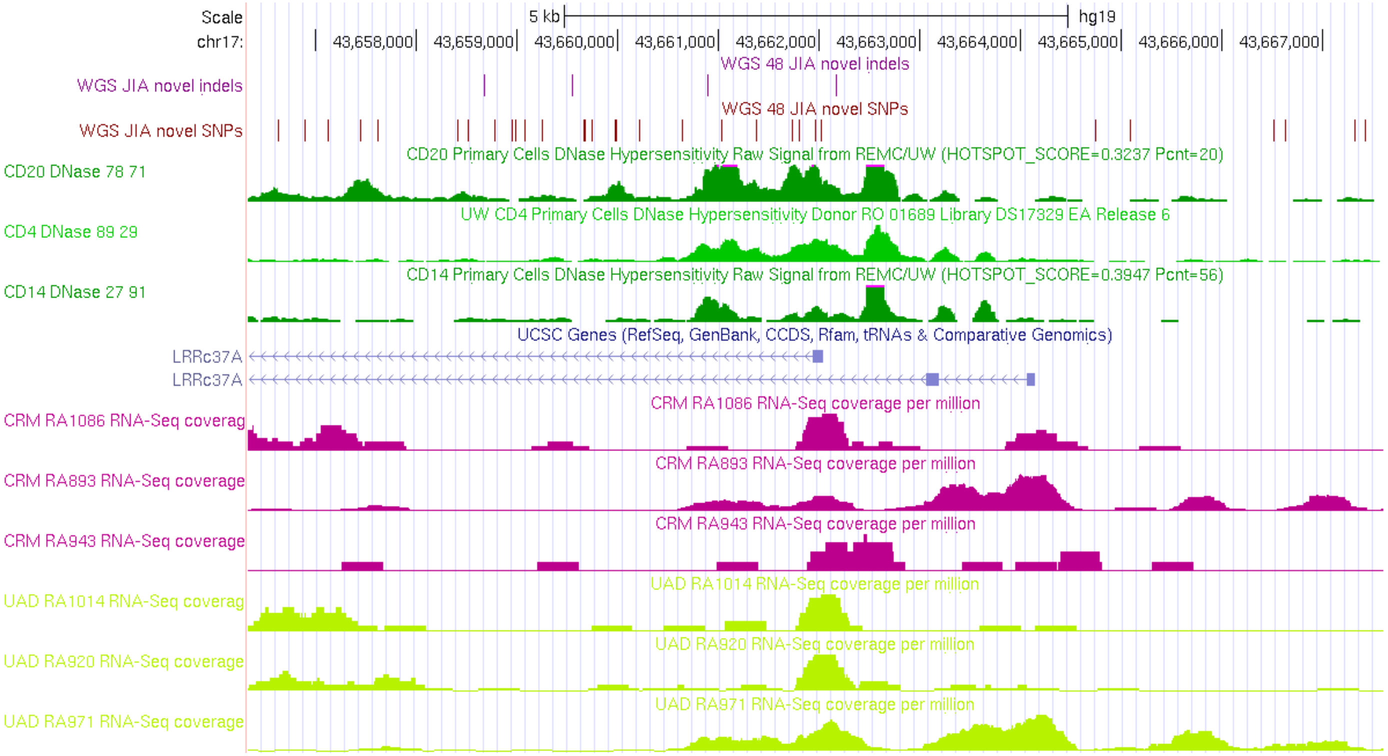

Supplementary Figure 9. Co-localization of JIA novel SNPs/indels with DNase hypersensitivity sites identified in CD20+ B cells, CD4+ T cells, and CD14+ monocytes. Genome browser view showing a genomic region on chromosome 17. This region contains novel JIA SNPs/indels overlapping with DNase hypersensitivity sites and JIA neutrophil transcriptome signals. UAD: children with untreated, active JIA and CRM: children who have achieved remission but are on medication.

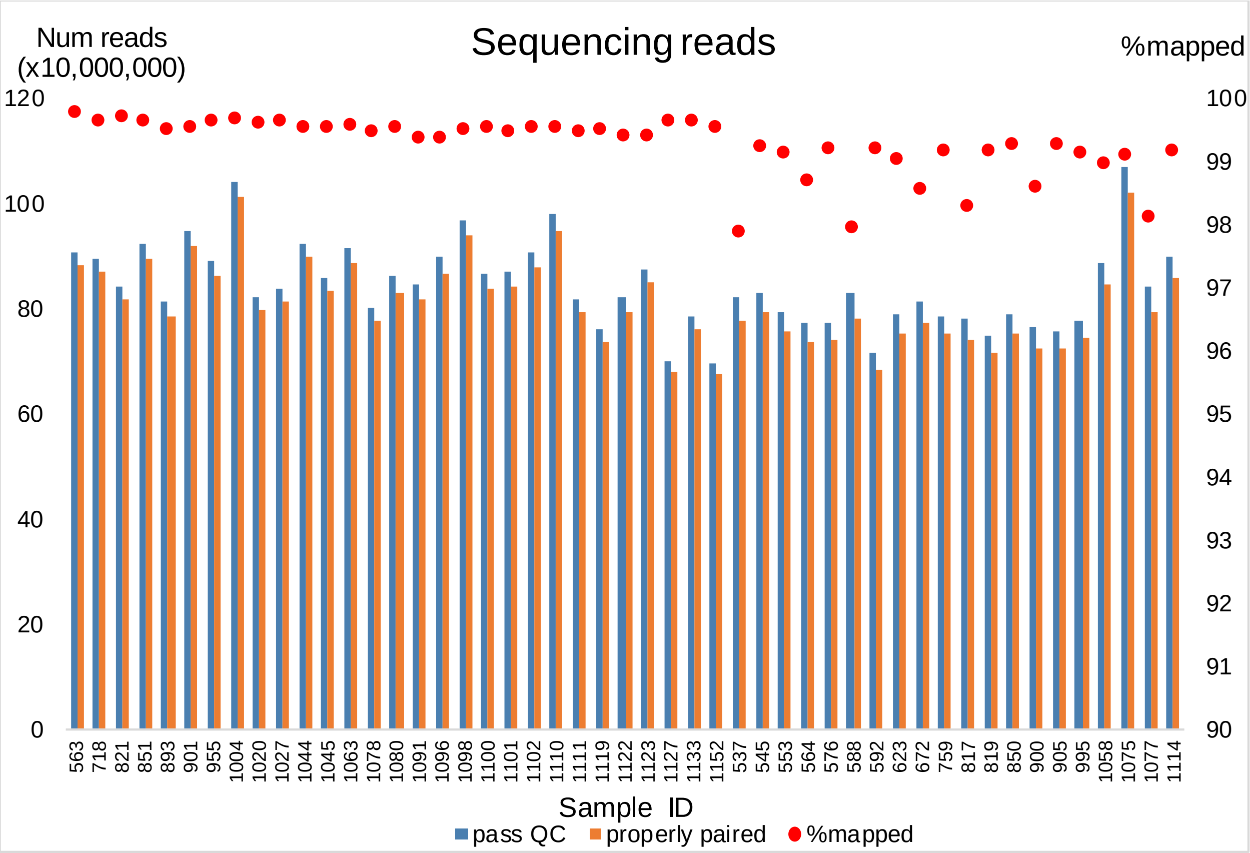

Supplementary Figure 10. Whole genome DNA sequencing reads. Blue bars represent pass quality control sequencing reads and orange bars indicate properly paired reads for each sample. Red dots show the percentage of mapped reads.

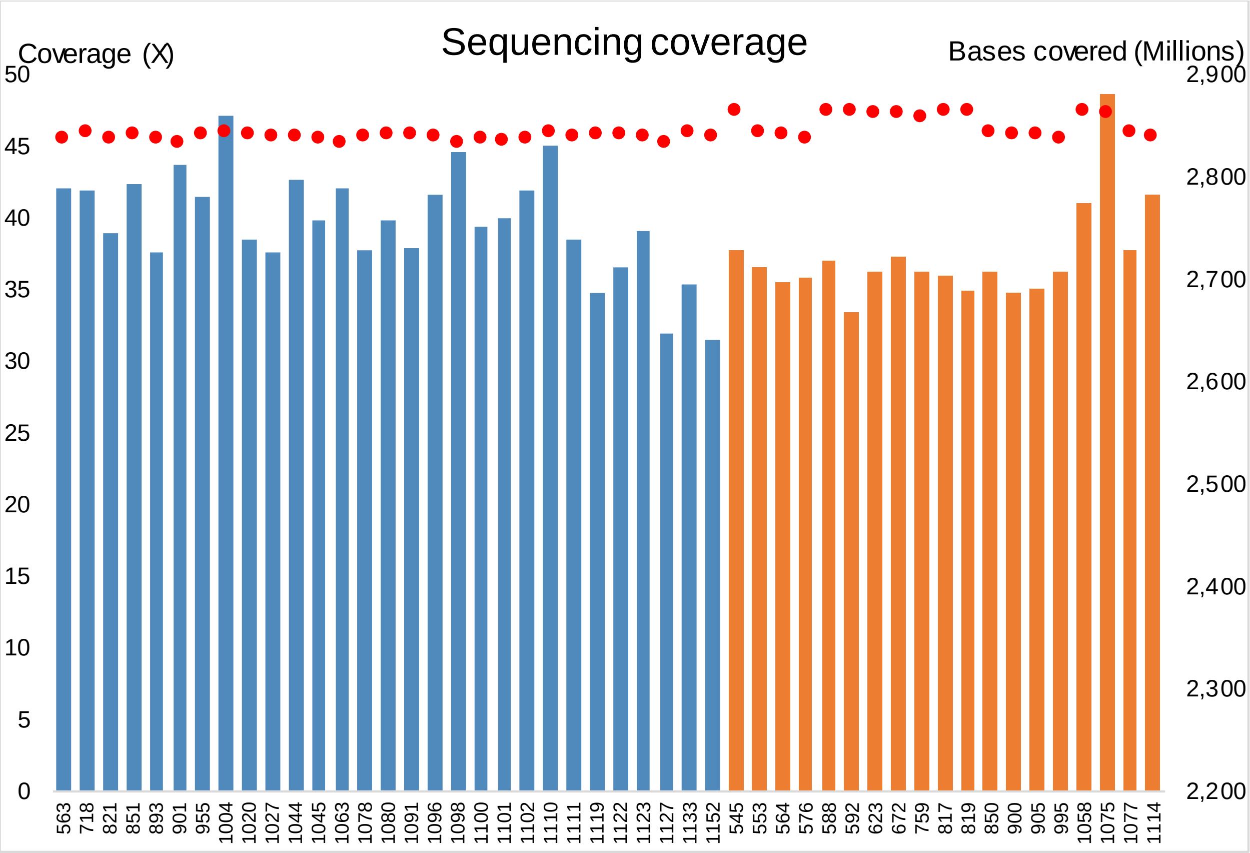

Supplementary Figure 11. Sequencing coverage. Bars indicate sequencing coverage for each sample (blue: batch 1 samples, orange: batch 2 samples). Red dots represent the number of bases covered in millions.

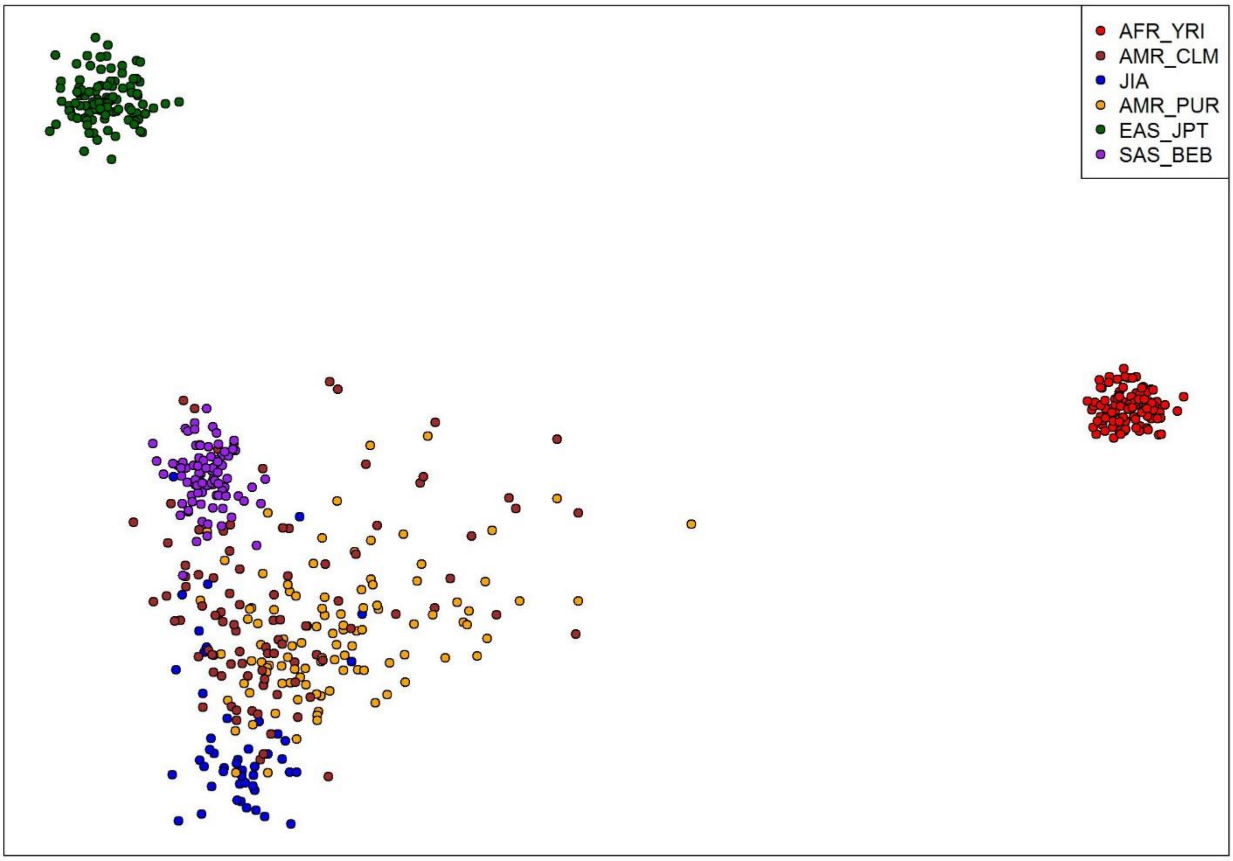

Supplementary Figure 12. Principal component analysis (PCA) of SNP genotypes for 48 JIA individuals and 1000 genomes samples. AFR_YRI: African, Yoruba in Ibadan, Nigeria; AMR_CLM: American, Colombian in Medellin, Colombia; JIA: American, Juvenile Idiopathic Arthritis; AMR_PUR: American Puerto Rican in Puerto Rico; EAS_JPT: East Asian, Japanese in Tokyo, Japan; SAS_BEB: South Asian, Bengali in Bangladesh.

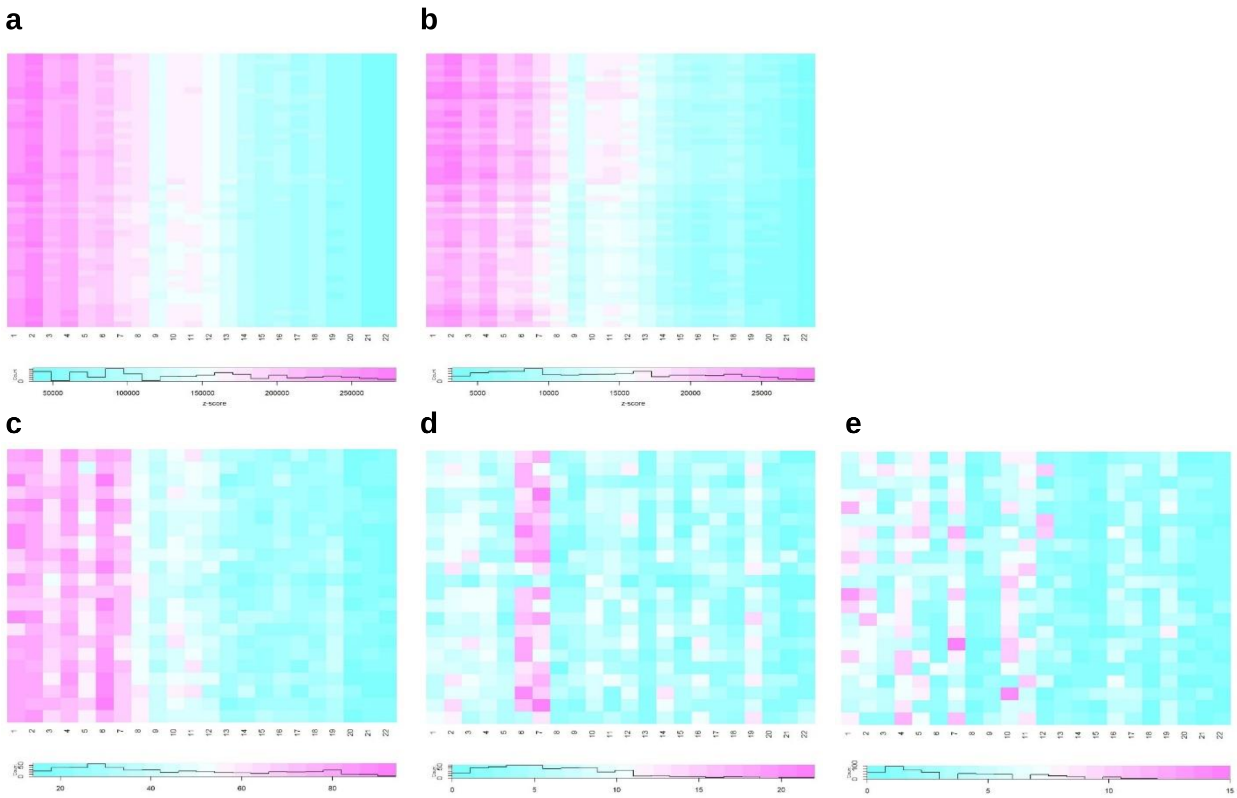

Supplementary Figure 13. Heatmap showing the number of variations discovered from deep whole genome DNA sequencing on 48 JIA individuals for (a) SNPs, (a) indels, (c) structural variation deletions, (d) structural variation duplications, (e) structural variation inversions. Rows represent samples and columns designate chromosome number.

Supplementary Table

Supplementary Table 1. Biological replicates: variants discovery concordance rates

	shared	biological replicate1 only	biological replicate2 only
SNP	3,549,146	43,345	51,466
concordance rate		98.79	98.57
indel	628,150	131,461	101,062
concordance rate		82.69	86.14

Supplementary Table 2. SNPs discovered from whole genome sequencing on 48 JIA individuals

	known		novel		All
Genomic feature	AAF>0.05	AAF≤0.05	AAF>0.05	AAF≤0.05	
DOWNSTREAM	468,891	282,909	3,238	96,107	851,145
EXON	19,371	11,156	161	3,697	34,385
INTERGENIC	2,340,329	1,331,783	9,852	432,441	4,114,405
INTRAGENIC	462	336	8	93	899
INTRON	1,447,573	881,141	4,540	289,045	2,622,299
MOTIF	2,849	2,145	30	884	5,908
NEXT_PROT	976,459	605,090	2,126	194,987	1,778,662
NON_SYN	19,342	22,744	203	4,686	46,975
SPLICE_SITE	7,695	5,683	53	1,492	14,923
START_GAINED	2,570	1,978	17	809	5,374
START_LOST	64	72		21	157
STOP_GAINED	200	348	3	148	699
STOP_LOST	48	42		14	104
SYNONYMOUS	19,644	16,094	119	2,208	38,065
UPSTREAM	633,558	387,071	5,130	136,737	1,162,496
UTR_3_PRIME	51,400	35,203	254	12,305	99,162
UTR_5_PRIME	12,541	8,233	72	3,717	24,563
Total	6,002,996	3,592,028	25,806	1,179,391	10,800,221

AAF: alternative allele frequency

Supplementary Table 3. Indels discovered from whole genome sequencing on 48 JIA individuals

	known		novel		All
Genomic feature	AAF>0.05	AAF≤0.05	AAF>0.05	AAF≤0.05	
DOWNSTREAM	49,525	25,265	3,367	21,784	99,941
EXON	1,253	609	67	471	2,400
INTERGENIC	225,045	105,449	12,512	86,004	429,010
INTRAGENIC	42	31	2	16	91
INTRON	146,035	73,863	7,554	61,902	289,354
MOTIF	447	211	53	251	962
NEXT_PROT	100,321	51,771	5,201	43,573	200,866
SPLICE_SITE	985	535	58	464	2,042
START_LOST	1	1		1	3
STOP_LOST	2			1	3
UPSTREAM	65,370	34,129	5,006	29,895	134,400
UTR_3_PRIME	6,803	3,661	342	3,169	13,975
UTR_5_PRIME	1,232	646	123	738	2,739
CODON_CHANGE	348	192	59	405	1,004
FRAME_SHIFT	406	234	74	462	1,176
Total	597,815	296,597	34,418	249,136	1,177,966

AAF: alternative allele frequency

Supplementary Table 4. Loss of Function (LoF) variants discovered from whole genome DNA sequencing on 48 JIA individuals

SNP	known	novel	Total (known+novel)
Non synonymous	42,086	4,890	46,976
start lost	136	21	157
stop gained	548	151	699
stop lost	90	12	102
splice site	13,378	1,544	14,922
Total	56,238	6,618	62,856
INDEL			
frameshift	640	536	1,176
splice site	1,525	517	2,042
Total	2,165	1,053	3,218

Supplementary Table 5. Regulation evidence of Loss of Function (LoF) variants with predicted damaging impact

	SNP			INDEL		
regulomeDB class	known	novel	all	known	novel	all
1a	13	0	13	0	0	0
1b	59	0	59	0	0	0
1c	3	0	3	0	0	0
1d	62	0	62	0	0	0
1e	0	0	0	0	0	0
1f	787	8	795	2	0	2
2a	203	2	205	16	3	19
2b	1,947	21	1,968	156	20	176
2c	63	0	63	4	0	4
3a	1,399	25	1,424	35	19	54
3b	7	0	7	0	4	4
4	6,820	150	6,970	275	63	338
5	15,098	139	15,237	680	112	792
6	4,300	24	4,324	600	75	675
Total	30,761	369	31,130	1,768	296	2,064

Supplementary Table 6. Classification of genetic regulation evidences base on RegulomeDB

Class	Regulatory evidences	JIA SNPs
1a	eQTL + TF binding + matched TF motif + matched DNase Footprint + DNase peak	628
1b	eQTL + TF binding + any motif + DNase Footprint + DNase peak	3,789
1c	eQTL + TF binding + matched TF motif + DNase peak	187
1d	eQTL + TF binding + any motif + DNase peak	3,595
1e	eQTL + TF binding + matched TF motif	52
1f	eQTL + TF binding / DNase peak	43,007
2a	TF binding + matched TF motif + matched DNase Footprint + DNase peak	18,569
2b	TF binding + any motif + DNase Footprint + DNase peak	186,331
2c	TF binding + matched TF motif + DNase peak	11,633
3a	TF binding + any motif + DNase peak	230,814
3b	TF binding + matched TF motif	5,129
4	TF binding + DNase peak	673,836
5	TF binding or DNase peak	2,286,082

Supplementary Table 7. Biological processes gene ontology annotation for genes of SNPs with class 1 regulation evidence

GO Term	Description	FDR q-value	Enrichment
GO:0044710	single-organism metabolic process	1.81E-23	2.47
GO:0044281	small molecule metabolic process	6.36E-22	3.33
GO:0019752	carboxylic acid metabolic process	3.88E-20	4.62
GO:0043436	oxoacid metabolic process	3.97E-20	4.6
GO:0071616	acyl-CoA biosynthetic process	9.40E-20	47.6
GO:0035384	thioester biosynthetic process	7.83E-20	47.6
GO:0006082	organic acid metabolic process	2.46E-19	4.28
GO:0006637	acyl-CoA metabolic process	6.98E-19	37.27
GO:0035383	thioester metabolic process	6.21E-19	37.27
GO:0032787	monocarboxylic acid metabolic process	1.82E-18	5.59
GO:0006631	fatty acid metabolic process	2.40E-18	14.25
GO:0035338	long-chain fatty-acyl-CoA biosynthetic process	1.21E-16	53.2
GO:0035337	fatty-acyl-CoA metabolic process	1.12E-16	53.2
GO:0035336	long-chain fatty-acyl-CoA metabolic process	1.04E-16	53.2
GO:0046949	fatty-acyl-CoA biosynthetic process	9.70E-17	53.2
GO:0006629	lipid metabolic process	1.04E-16	3.66
GO:0044711	single-organism biosynthetic process	2.17E-16	3.62
GO:0090662	ATP hydrolysis coupled transmembrane transport	5.24E-16	13.09
GO:0044255	cellular lipid metabolic process	9.94E-16	3.91
GO:0015988	energy coupled proton transmembrane transport, against electrochemical gradient	7.60E-14	13
GO:0015991	ATP hydrolysis coupled proton transport	7.23E-14	13
GO:0009108	coenzyme biosynthetic process	4.15E-13	21.16
GO:0044272	sulfur compound biosynthetic process	8.29E-12	14.42
GO:0033700	phospholipid efflux	1.32E-11	19.91
GO:0055114	oxidation-reduction process	1.30E-11	3.57
GO:0051188	cofactor biosynthetic process	1.70E-11	15.87
GO:0051056	regulation of small GTPase mediated signal transduction	1.64E-11	4.48
GO:0008152	metabolic process	2.71E-11	1.45
GO:0006869	lipid transport	2.98E-11	4.29
GO:0044699	single-organism process	3.06E-11	1.22
GO:0044763	single-organism cellular process	4.33E-11	1.28
GO:0006790	sulfur compound metabolic process	4.97E-11	9.47
GO:1901135	carbohydrate derivative metabolic process	5.36E-11	2.53

GO:0051345	positive regulation of hydrolase activity	5.87E-11	2.65
GO:0006732	coenzyme metabolic process	8.70E-11	11.45
GO:0043087	regulation of GTPase activity	9.67E-11	2.85
GO:0043547	positive regulation of GTPase activity	1.33E-10	3.03
GO:0055085	transmembrane transport	2.62E-10	9.02
GO:1901137	carbohydrate derivative biosynthetic process	3.49E-10	3.23
GO:0015914	phospholipid transport	9.17E-10	7.93
GO:0071704	organic substance metabolic process	9.62E-10	1.45
GO:0051186	cofactor metabolic process	1.20E-09	5.18
GO:0033539	fatty acid beta-oxidation using acyl-CoA dehydrogenase	1.38E-09	69.69
GO:0043085	positive regulation of catalytic activity	2.39E-09	2.14
GO:0006635	fatty acid beta-oxidation	3.75E-09	35.84
GO:0044712	single-organism catabolic process	1.02E-08	2.94
GO:0035023	regulation of Rho protein signal transduction	1.98E-08	5.54
GO:0044238	primary metabolic process	2.12E-08	1.42
GO:0044093	positive regulation of molecular function	2.12E-08	1.98
GO:0097035	regulation of membrane lipid distribution	2.12E-08	9.86
GO:0044242	cellular lipid catabolic process	2.53E-08	16.15
GO:0019395	fatty acid oxidation	2.56E-08	10.84
GO:0072522	purine-containing compound biosynthetic process	2.62E-08	5.1
GO:0034308	primary alcohol metabolic process	2.92E-08	18.54
GO:0033344	cholesterol efflux	3.13E-08	13.69
GO:0015748	organophosphate ester transport	3.19E-08	6.33
GO:0046033	AMP metabolic process	3.40E-08	23.87
GO:0016053	organic acid biosynthetic process	3.34E-08	5.67
GO:0034440	lipid oxidation	3.41E-08	10.54
GO:0044282	small molecule catabolic process	4.32E-08	4.56
GO:0090383	phagosome acidification	4.49E-08	10.34
GO:0009152	purine ribonucleotide biosynthetic process	6.07E-08	5.32
GO:0071702	organic substance transport	7.64E-08	1.78
GO:0009127	purine nucleoside monophosphate biosynthetic process	8.11E-08	6.81
GO:0009168	purine ribonucleoside monophosphate biosynthetic process	7.98E-08	6.81
GO:0071827	plasma lipoprotein particle organization	7.99E-08	11.38
GO:0006164	purine nucleotide biosynthetic process	8.32E-08	5.23
GO:0009062	fatty acid catabolic process	1.11E-07	24.89

GO:0046394	carboxylic acid biosynthetic process	1.16E-07	5.85
GO:0051336	regulation of hydrolase activity	1.33E-07	2.06
GO:0001676	long-chain fatty acid metabolic process	1.39E-07	9.58
GO:0071825	protein-lipid complex subunit organization	1.55E-07	10.86
GO:0055088	lipid homeostasis	1.64E-07	19.15
GO:1902600	hydrogen ion transmembrane transport	2.06E-07	6.06
GO:1901564	organonitrogen compound metabolic process	2.19E-07	1.8
GO:0008610	lipid biosynthetic process	2.50E-07	3.55
GO:0072329	monocarboxylic acid catabolic process	2.86E-07	22.4
GO:0006818	hydrogen transport	3.45E-07	5.08
GO:0044283	small molecule biosynthetic process	3.83E-07	4.11
GO:0006067	ethanol metabolic process	5.35E-07	31.52
GO:0006419	alanyl-tRNA aminoacylation	5.87E-07	816.4
GO:0046390	ribose phosphate biosynthetic process	5.92E-07	4.75
GO:0009260	ribonucleotide biosynthetic process	5.85E-07	4.75
GO:0048878	chemical homeostasis	6.47E-07	2.1
GO:0042592	homeostatic process	7.48E-07	1.88
GO:0015992	proton transport	1.04E-06	5.01
GO:0030301	cholesterol transport	1.13E-06	8.1
GO:0046395	carboxylic acid catabolic process	1.35E-06	5.63
GO:0034377	plasma lipoprotein particle assembly	1.45E-06	15.93
GO:1901657	glycosyl compound metabolic process	1.94E-06	3.2
GO:0009156	ribonucleoside monophosphate biosynthetic process	1.96E-06	5.68
GO:0015918	sterol transport	2.02E-06	7.8
GO:0098656	anion transmembrane transport	2.06E-06	23.67
GO:0006810	transport	2.21E-06	1.48
GO:0032371	regulation of sterol transport	2.28E-06	9.96
GO:0032374	regulation of cholesterol transport	2.26E-06	9.96
GO:0006820	anion transport	2.33E-06	13.02
GO:0072330	monocarboxylic acid biosynthetic process	2.50E-06	6.56
GO:0016054	organic acid catabolic process	2.48E-06	5.13
GO:0008150	biological_process	2.49E-06	1.06
GO:0006081	cellular aldehyde metabolic process	2.53E-06	12.42
GO:0042451	purine nucleoside biosynthetic process	2.67E-06	5.26
GO:0046129	purine ribonucleoside biosynthetic process	2.65E-06	5.26

GO:0016042	lipid catabolic process	2.85E-06	10.3
GO:0009165	nucleotide biosynthetic process	2.87E-06	4.06
GO:0015850	organic hydroxy compound transport	2.85E-06	31.13
GO:0044765	single-organism transport	3.71E-06	4.18
GO:0043691	reverse cholesterol transport	4.09E-06	14.48
GO:0065005	protein-lipid complex assembly	4.05E-06	14.48
GO:0006639	acylglycerol metabolic process	4.24E-06	6.49
GO:0006172	ADP biosynthetic process	4.26E-06	36.94
GO:0009188	ribonucleoside diphosphate biosynthetic process	4.22E-06	36.94
GO:0009180	purine ribonucleoside diphosphate biosynthetic process	4.18E-06	36.94
GO:0009136	purine nucleoside diphosphate biosynthetic process	4.15E-06	36.94
GO:0009133	nucleoside diphosphate biosynthetic process	4.11E-06	36.94
GO:0044237	cellular metabolic process	4.14E-06	1.36
GO:1901293	nucleoside phosphate biosynthetic process	4.36E-06	3.96
GO:0030004	cellular monovalent inorganic cation homeostasis	4.37E-06	5.07
GO:0015949	nucleobase-containing small molecule interconversion	4.95E-06	21.26
GO:0006638	neutral lipid metabolic process	5.56E-06	6.35
GO:0045851	pH reduction	5.54E-06	7.52
GO:0033572	transferrin transport	5.50E-06	7.52
GO:0051452	intracellular pH reduction	5.45E-06	7.52
GO:0010872	regulation of cholesterol esterification	5.42E-06	19.91
GO:0051234	establishment of localization	5.63E-06	1.45
GO:0008286	insulin receptor signaling pathway	6.62E-06	4.83
GO:0006069	ethanol oxidation	7.54E-06	31.89
GO:0034310	primary alcohol catabolic process	7.94E-06	31.52
GO:0015682	ferric iron transport	9.99E-06	7.2
GO:0072512	trivalent inorganic cation transport	9.91E-06	7.2
GO:0009124	nucleoside monophosphate biosynthetic process	1.03E-05	5.07
GO:0034367	macromolecular complex remodeling	1.10E-05	11.2
GO:0034368	protein-lipid complex remodeling	1.09E-05	11.2
GO:0034369	plasma lipoprotein particle remodeling	1.09E-05	11.2
GO:0050790	regulation of catalytic activity	1.15E-05	1.7
GO:1902578	single-organism localization	1.28E-05	3.84
GO:0035608	protein deglutamylation	1.53E-05	34.19
GO:0006633	fatty acid biosynthetic process	1.68E-05	7.19

GO:0045332	phospholipid translocation	1.69E-05	10.92
GO:1901566	organonitrogen compound biosynthetic process	1.85E-05	1.99
GO:0019372	lipoxygenase pathway	2.03E-05	21.56
GO:0034220	ion transmembrane transport	2.09E-05	2.2
GO:0050994	regulation of lipid catabolic process	2.09E-05	8.15
GO:0015031	protein transport	2.25E-05	1.86
GO:0010875	positive regulation of cholesterol efflux	2.72E-05	96.05
GO:0016101	diterpenoid metabolic process	3.61E-05	7.03
GO:0042455	ribonucleoside biosynthetic process	4.07E-05	4.43
GO:0032373	positive regulation of sterol transport	4.06E-05	87.32
GO:0032376	positive regulation of cholesterol transport	4.04E-05	87.32
GO:0046578	regulation of Ras protein signal transduction	4.66E-05	3.56
GO:0009119	ribonucleoside metabolic process	4.72E-05	3.14
GO:0009142	nucleoside triphosphate biosynthetic process	5.20E-05	5.46
GO:0034204	lipid translocation	5.27E-05	9.93
GO:0042157	lipoprotein metabolic process	5.45E-05	7.52
GO:0045184	establishment of protein localization	5.78E-05	1.71
GO:0010874	regulation of cholesterol efflux	5.82E-05	80.04
GO:0010248	establishment or maintenance of transmembrane electrochemical gradient	6.18E-05	10.74
GO:0035610	protein side chain deglutamylation	6.43E-05	41.03
GO:0051179	localization	6.41E-05	1.38
GO:0055067	monovalent inorganic cation homeostasis	6.38E-05	4.28
GO:0007190	activation of adenylate cyclase activity	7.33E-05	21.73
GO:0070085	glycosylation	7.55E-05	2.92
GO:0099517	synaptic vesicle transport along microtubule	8.21E-05	16.33
GO:0099514	synaptic vesicle cytoskeletal transport	8.16E-05	16.33
GO:0048490	anterograde synaptic vesicle transport	8.11E-05	16.33
GO:0010873	positive regulation of cholesterol esterification	8.47E-05	19.91
GO:0034380	high-density lipoprotein particle assembly	8.45E-05	19.91
GO:0006101	citrate metabolic process	8.71E-05	110.32
GO:0006490	oligosaccharide-lipid intermediate biosynthetic process	9.31E-05	18.08
GO:0006488	dolichol-linked oligosaccharide biosynthetic process	9.25E-05	18.08
GO:0043687	post-translational protein modification	9.96E-05	7.41
GO:0006486	protein glycosylation	1.04E-04	2.93
GO:0043413	macromolecule glycosylation	1.03E-04	2.93

GO:0033540	fatty acid beta-oxidation using acyl-CoA oxidase	1.05E-04	56.89
GO:0065008	regulation of biological quality	1.11E-04	1.41
GO:0009116	nucleoside metabolic process	1.11E-04	2.94
GO:0006171	cAMP biosynthetic process	1.18E-04	44.43
GO:0016024	CDP-diacylglycerol biosynthetic process	1.21E-04	26.17
GO:0044598	doxorubicin metabolic process	1.30E-04	34.02
GO:0044597	daunorubicin metabolic process	1.29E-04	34.02
GO:0030819	positive regulation of cAMP biosynthetic process	1.30E-04	9.68
GO:0009163	nucleoside biosynthetic process	1.33E-04	4.07
GO:0035510	DNA dealkylation	1.39E-04	11.1
GO:0001523	retinoid metabolic process	1.47E-04	6.84
GO:0006958	complement activation, classical pathway	1.50E-04	5.17
GO:0055086	nucleobase-containing small molecule metabolic process	1.68E-04	2.4
GO:1901659	glycosyl compound biosynthetic process	1.67E-04	4.01
GO:0072521	purine-containing compound metabolic process	1.72E-04	2.82
GO:0006721	terpenoid metabolic process	2.19E-04	5.97
GO:0090407	organophosphate biosynthetic process	2.31E-04	3.54
GO:2001300	lipoxin metabolic process	2.37E-04	28.85
GO:0051122	hepoxilin biosynthetic process	2.41E-04	28.75
GO:0051121	hepoxilin metabolic process	2.40E-04	28.75
GO:0048513	animal organ development	2.44E-04	1.73
GO:0046341	CDP-diacylglycerol metabolic process	2.53E-04	23.26
GO:0042493	response to drug	2.75E-04	10.53
GO:0006826	iron ion transport	2.83E-04	5.52
GO:0035280	miRNA loading onto RISC involved in gene silencing by miRNA	2.96E-04	34.37
GO:0000041	transition metal ion transport	2.95E-04	4.24
GO:0036376	sodium ion export from cell	3.28E-04	14.37
GO:0045762	positive regulation of adenylate cyclase activity	3.36E-04	12.42
GO:0006066	alcohol metabolic process	3.45E-04	3.51
GO:0016137	glycoside metabolic process	3.47E-04	21.26
GO:0009126	purine nucleoside monophosphate metabolic process	3.51E-04	3.52
GO:0009167	purine ribonucleoside monophosphate metabolic process	3.50E-04	3.52
GO:0098660	inorganic ion transmembrane transport	3.86E-04	2.26
GO:0010528	regulation of transposition	4.33E-04	16.88
GO:0010529	negative regulation of transposition	4.31E-04	16.88

GO:0006216	cytidine catabolic process	4.29E-04	16.88
GO:0046087	cytidine metabolic process	4.27E-04	16.88
GO:0009972	cytidine deamination	4.25E-04	16.88
GO:0030816	positive regulation of cAMP metabolic process	4.25E-04	8.41
GO:0045834	positive regulation of lipid metabolic process	4.50E-04	4.53
GO:0006796	phosphate-containing compound metabolic process	4.71E-04	1.86
GO:0009066	aspartate family amino acid metabolic process	4.70E-04	6.42
GO:0008104	protein localization	4.87E-04	1.57
GO:0006520	cellular amino acid metabolic process	4.91E-04	2.92
GO:0009150	purine ribonucleotide metabolic process	5.08E-04	2.89
GO:0032368	regulation of lipid transport	5.18E-04	5.08
GO:0018146	keratan sulfate biosynthetic process	5.23E-04	6.75
GO:0006793	phosphorus metabolic process	5.38E-04	1.85
GO:0010738	regulation of protein kinase A signaling	5.41E-04	19.72
GO:0065009	regulation of molecular function	5.64E-04	1.52
GO:0033036	macromolecule localization	5.92E-04	1.56
GO:0009991	response to extracellular stimulus	6.05E-04	2.21
GO:0030641	regulation of cellular pH	6.17E-04	4.36
GO:0030258	lipid modification	6.78E-04	8.53
GO:0007189	adenylate cyclase-activating G-protein coupled receptor signaling pathway	6.79E-04	21.13
GO:0030638	polyketide metabolic process	6.90E-04	27.21
GO:0030647	aminoglycoside antibiotic metabolic process	6.87E-04	27.21
GO:0034375	high-density lipoprotein particle remodeling	7.65E-04	11.95
GO:0006641	triglyceride metabolic process	7.64E-04	5.76
GO:0030804	positive regulation of cyclic nucleotide biosynthetic process	7.72E-04	7.79
GO:0008202	steroid metabolic process	7.98E-04	3.7
GO:0015985	energy coupled proton transport, down electrochemical gradient	8.37E-04	10.52
GO:0015986	ATP synthesis coupled proton transport	8.34E-04	10.52
GO:0070922	small RNA loading onto RISC	8.73E-04	28.65
GO:0098662	inorganic cation transmembrane transport	1.03E-03	2.29
GO:0009141	nucleoside triphosphate metabolic process	1.09E-03	3.27
GO:0080111	DNA demethylation	1.19E-03	14.54
GO:0046128	purine ribonucleoside metabolic process	1.19E-03	2.96
GO:0009161	ribonucleoside monophosphate metabolic process	1.26E-03	3.23
GO:0046034	ATP metabolic process	1.27E-03	3.58

GO:0042759	long-chain fatty acid biosynthetic process	1.27E-03	23
GO:0006508	proteolysis	1.28E-03	2.51
GO:0010745	negative regulation of macrophage derived foam cell differentiation	1.31E-03	80.04
GO:0009605	response to external stimulus	1.34E-03	1.61
GO:0008652	cellular amino acid biosynthetic process	1.43E-03	5.71
GO:0007584	response to nutrient	1.44E-03	4.46
GO:0006956	complement activation	1.45E-03	4.31
GO:0006163	purine nucleotide metabolic process	1.46E-03	2.72
GO:0009259	ribonucleotide metabolic process	1.46E-03	2.72
GO:0042339	keratan sulfate metabolic process	1.46E-03	6.04
GO:0006885	regulation of pH	1.50E-03	4.04
GO:0008088	axo-dendritic transport	1.51E-03	7.51
GO:0032370	positive regulation of lipid transport	1.54E-03	35.57
GO:0043268	positive regulation of potassium ion transport	1.58E-03	6.16
GO:1900373	positive regulation of purine nucleotide biosynthetic process	1.59E-03	7.1
GO:0030810	positive regulation of nucleotide biosynthetic process	1.58E-03	7.1
GO:0018410	C-terminal protein amino acid modification	1.60E-03	9.88
GO:0044804	nucleophagy	1.59E-03	9.88
GO:0042981	regulation of apoptotic process	1.60E-03	1.57
GO:0042985	negative regulation of amyloid precursor protein biosynthetic process	1.61E-03	259.17
GO:0006084	acetyl-CoA metabolic process	1.62E-03	30.29
GO:0042278	purine nucleoside metabolic process	1.62E-03	2.91
GO:0009083	branched-chain amino acid catabolic process	1.77E-03	7.57
GO:0009081	branched-chain amino acid metabolic process	1.77E-03	7.57
GO:0006023	aminoglycan biosynthetic process	1.78E-03	3.64
GO:0031281	positive regulation of cyclase activity	1.79E-03	9.56
GO:0016241	regulation of macroautophagy	1.79E-03	3.42
GO:0008637	apoptotic mitochondrial changes	1.80E-03	4.42
GO:0030801	positive regulation of cyclic nucleotide metabolic process	1.84E-03	6.94
GO:0030817	regulation of cAMP biosynthetic process	1.83E-03	6.94
GO:0055092	sterol homeostasis	1.87E-03	6.4
GO:0042632	cholesterol homeostasis	1.87E-03	6.4
GO:0033559	unsaturated fatty acid metabolic process	1.94E-03	5.99
GO:0030003	cellular cation homeostasis	1.96E-03	2.09
GO:0051453	regulation of intracellular pH	2.02E-03	4.16

GO:0006720	isoprenoid metabolic process	2.05E-03	4.78
GO:0019432	triglyceride biosynthetic process	2.11E-03	16.1
GO:0031667	response to nutrient levels	2.11E-03	2.14
GO:0007420	brain development	2.12E-03	2.85
GO:0030104	water homeostasis	2.12E-03	6.28
GO:0033993	response to lipid	2.18E-03	3.89
GO:0048871	multicellular organismal homeostasis	2.17E-03	5.84
GO:0051349	positive regulation of lyase activity	2.26E-03	9.2
GO:0042574	retinal metabolic process	2.34E-03	20.69
GO:0043067	regulation of programmed cell death	2.36E-03	1.56
GO:0006501	C-terminal protein lipidation	2.40E-03	14.82
GO:0006068	ethanol catabolic process	2.55E-03	31.52
GO:0006083	acetate metabolic process	2.56E-03	31.4
GO:0009064	glutamine family amino acid metabolic process	2.65E-03	4.84
GO:0046133	pyrimidine ribonucleoside catabolic process	2.64E-03	12.66
GO:0009144	purine nucleoside triphosphate metabolic process	2.70E-03	3.12
GO:0008089	anterograde axonal transport	2.69E-03	10.05
GO:0055082	cellular chemical homeostasis	2.74E-03	1.97
GO:0046460	neutral lipid biosynthetic process	2.78E-03	10.53
GO:0046463	acylglycerol biosynthetic process	2.77E-03	10.53
GO:0045761	regulation of adenylate cyclase activity	2.78E-03	8.87
GO:0055080	cation homeostasis	2.84E-03	1.98
GO:0006418	tRNA aminoacylation for protein translation	2.87E-03	69.98
GO:0060191	regulation of lipase activity	2.91E-03	4.95
GO:1903792	negative regulation of anion transport	2.92E-03	9.28
GO:0019693	ribose phosphate metabolic process	2.94E-03	2.6
GO:0019217	regulation of fatty acid metabolic process	2.97E-03	5.41
GO:0046939	nucleotide phosphorylation	2.96E-03	7.28
GO:0052652	cyclic purine nucleotide metabolic process	3.04E-03	13.43
GO:0097502	mannosylation	3.03E-03	13.69
GO:0006873	cellular ion homeostasis	3.10E-03	2.04
GO:0001973	adenosine receptor signaling pathway	3.14E-03	38.51
GO:1901615	organic hydroxy compound metabolic process	3.15E-03	2.7
GO:0009151	purine deoxyribonucleotide metabolic process	3.17E-03	20.83
GO:0098655	cation transmembrane transport	3.17E-03	2.13

GO:0031054	pre-miRNA processing	3.19E-03	21.48
GO:0009123	nucleoside monophosphate metabolic process	3.36E-03	2.99
GO:0006591	ornithine metabolic process	3.51E-03	13.17
GO:0015672	monovalent inorganic cation transport	3.54E-03	2.4
GO:0043038	amino acid activation	3.53E-03	64.45
GO:0043039	tRNA aminoacylation	3.52E-03	64.45
GO:0032367	intracellular cholesterol transport	3.59E-03	160.08
GO:0036109	alpha-linolenic acid metabolic process	3.76E-03	44.37
GO:0042454	ribonucleoside catabolic process	3.76E-03	9.35
GO:0038027	apolipoprotein A-I-mediated signaling pathway	3.91E-03	194.38
GO:0072350	tricarboxylic acid metabolic process	3.93E-03	47.28
GO:0010896	regulation of triglyceride catabolic process	3.93E-03	16.01
GO:1901565	organonitrogen compound catabolic process	4.12E-03	2.65
GO:0009132	nucleoside diphosphate metabolic process	4.32E-03	6.05
GO:0071397	cellular response to cholesterol	4.46E-03	204.1
GO:0009117	nucleotide metabolic process	4.51E-03	3.24
GO:0090066	regulation of anatomical structure size	4.53E-03	2.07
GO:0010561	negative regulation of glycoprotein biosynthetic process	4.54E-03	44.29
GO:0071436	sodium ion export	4.54E-03	10.27
GO:0010107	potassium ion import	4.53E-03	10.27
GO:0030007	cellular potassium ion homeostasis	4.52E-03	10.27
GO:0010941	regulation of cell death	4.65E-03	1.52
GO:0098771	inorganic ion homeostasis	4.68E-03	1.93
GO:0046031	ADP metabolic process	4.69E-03	8.02
GO:0099518	vesicle cytoskeletal trafficking	4.68E-03	7.62
GO:0009067	aspartate family amino acid biosynthetic process	4.68E-03	7.61
GO:0006024	glycosaminoglycan biosynthetic process	4.70E-03	3.47
GO:0009205	purine ribonucleoside triphosphate metabolic process	4.74E-03	3.07
GO:0009190	cyclic nucleotide biosynthetic process	4.81E-03	12.21
GO:0030799	regulation of cyclic nucleotide metabolic process	4.88E-03	5.38
GO:0006690	icosanoid metabolic process	4.88E-03	5.88
GO:0061591	calcium activated galactosylceramide scrambling	5.05E-03	23.6
GO:0061588	calcium activated phospholipid scrambling	5.04E-03	23.6
GO:0061589	calcium activated phosphatidylserine scrambling	5.02E-03	23.6
GO:0061590	calcium activated phosphatidylcholine scrambling	5.01E-03	23.6

GO:0050801	ion homeostasis	5.02E-03	1.92
GO:0050995	negative regulation of lipid catabolic process	5.10E-03	11.12
GO:0051004	regulation of lipoprotein lipase activity	5.09E-03	11.12
GO:0006754	ATP biosynthetic process	5.30E-03	5.91
GO:0006753	nucleoside phosphate metabolic process	5.46E-03	3.18
GO:0006654	phosphatidic acid biosynthetic process	5.49E-03	13.08
GO:0043266	regulation of potassium ion transport	5.75E-03	5.62
GO:1901568	fatty acid derivative metabolic process	6.02E-03	16.48
GO:0043651	linoleic acid metabolic process	6.03E-03	16.43
GO:0050996	positive regulation of lipid catabolic process	6.02E-03	8.6
GO:0010942	positive regulation of cell death	6.19E-03	1.8
GO:0030802	regulation of cyclic nucleotide biosynthetic process	6.18E-03	5.81
GO:0030814	regulation of cAMP metabolic process	6.17E-03	5.81
GO:0045940	positive regulation of steroid metabolic process	6.19E-03	8.53
GO:0006883	cellular sodium ion homeostasis	6.33E-03	7.84
GO:0030100	regulation of endocytosis	6.35E-03	2.26
GO:0042984	regulation of amyloid precursor protein biosynthetic process	6.37E-03	155.5
GO:0033211	adiponectin-activated signaling pathway	6.58E-03	32.02
GO:0015711	organic anion transport	6.69E-03	10.78
GO:0032929	negative regulation of superoxide anion generation	6.81E-03	97.19
GO:1903019	negative regulation of glycoprotein metabolic process	6.79E-03	38.75
GO:0015718	monocarboxylic acid transport	6.85E-03	14.29
GO:0044723	single-organism carbohydrate metabolic process	6.86E-03	1.92
GO:0044248	cellular catabolic process	6.86E-03	2.9
GO:0006085	acetyl-CoA biosynthetic process	6.89E-03	35.7
GO:0046473	phosphatidic acid metabolic process	7.01E-03	12.31
GO:0070383	DNA cytosine deamination	7.41E-03	20.26
GO:0010898	positive regulation of triglyceride catabolic process	7.50E-03	20.06
GO:0009199	ribonucleoside triphosphate metabolic process	7.54E-03	2.95
GO:0071875	adrenergic receptor signaling pathway	7.59E-03	26.86
GO:0010901	regulation of very-low-density lipoprotein particle remodeling	7.61E-03	20.01
GO:0010916	negative regulation of very-low-density lipoprotein particle clearance	7.59E-03	20.01
GO:0010915	regulation of very-low-density lipoprotein particle clearance	7.57E-03	20.01
GO:0032489	regulation of Cdc42 protein signal transduction	7.61E-03	19.91
GO:0032365	intracellular lipid transport	7.64E-03	39.76

GO:0043065	positive regulation of apoptotic process	7.66E-03	1.79
GO:0006811	ion transport	8.22E-03	4.76
GO:0019216	regulation of lipid metabolic process	8.41E-03	2.93
GO:0036315	cellular response to sterol	8.56E-03	145.79
GO:0098930	axonal transport	8.62E-03	8.16
GO:0032366	intracellular sterol transport	8.64E-03	120.06
GO:0055091	phospholipid homeostasis	8.62E-03	120.06
GO:0043068	positive regulation of programmed cell death	8.60E-03	1.78
GO:0031279	regulation of cyclase activity	8.63E-03	7.31
GO:0019542	propionate biosynthetic process	8.69E-03	83.31
GO:0019541	propionate metabolic process	8.67E-03	83.31
GO:0019413	acetate biosynthetic process	8.65E-03	83.31
GO:0019427	acetyl-CoA biosynthetic process from acetate	8.62E-03	83.31
GO:0046461	neutral lipid catabolic process	8.97E-03	7.96
GO:0046464	acylglycerol catabolic process	8.94E-03	7.96
GO:0019725	cellular homeostasis	9.10E-03	1.88
GO:0034637	cellular carbohydrate biosynthetic process	9.18E-03	4.82
GO:0038034	signal transduction in absence of ligand	9.21E-03	5.18
GO:0097192	extrinsic apoptotic signaling pathway in absence of ligand	9.19E-03	5.18
GO:0051402	neuron apoptotic process	9.17E-03	5.18
GO:0010559	regulation of glycoprotein biosynthetic process	9.17E-03	18.99
GO:0071830	triglyceride-rich lipoprotein particle clearance	9.32E-03	13.27
GO:0034382	chylomicron remnant clearance	9.29E-03	13.27
GO:0009145	purine nucleoside triphosphate biosynthetic process	9.52E-03	4.86
GO:0019637	organophosphate metabolic process	9.56E-03	2.24
GO:0045981	positive regulation of nucleotide metabolic process	9.56E-03	5.41
GO:1900544	positive regulation of purine nucleotide metabolic process	9.54E-03	5.41
GO:0060192	negative regulation of lipase activity	9.87E-03	8.14

Supplementary Table 8. SNPs hotspot regions

Chromosome	Start position	End position
1	148000000	149000000
2	91000000	92000000
6	29000000	30000000
6	29000000	30000000
6	30000000	31000000
6	31000000	32000000
6	32000000	33000000
6	33000000	34000000
8	7000000	8000000
9	40000000	41000000
9	41000000	42000000
9	42000000	43000000
9	45000000	46000000
9	46000000	47000000
9	65000000	66000000
9	67000000	68000000
9	69000000	70000000
14	19000000	20000000

Supplementary Table 9. Biological processes gene ontology annotation for gene associated with SNP hotspots

GO Term	Description	FDR q-value	Enrichment
GO:0050863	regulation of T cell activation	4.78E-02	2.36
GO:0045087	innate immune response	3.28E-02	2.75
GO:0009605	response to external stimulus	3.40E-02	2.54
GO:0002696	positive regulation of leukocyte activation	2.69E-02	2.36
GO:0050870	positive regulation of T cell activation	2.15E-02	2.36
GO:0050867	positive regulation of cell activation	1.79E-02	2.36
GO:0051251	positive regulation of lymphocyte activation	1.54E-02	2.36
GO:0002504	antigen processing and presentation of peptide or polysaccharide antigen via MHC class II	2.18E-02	2.44
GO:0002495	antigen processing and presentation of peptide antigen via MHC class II	1.94E-02	2.44
GO:0019886	antigen processing and presentation of exogenous peptide antigen via MHC class II	1.75E-02	2.44
GO:0002376	immune system process	1.82E-02	1.6
GO:0002694	regulation of leukocyte activation	3.12E-02	1.89
GO:0050865	regulation of cell activation	2.88E-02	1.89
GO:0051249	regulation of lymphocyte activation	2.67E-02	1.89
GO:0006954	inflammatory response	3.37E-02	7.86
GO:0006952	defense response	3.30E-02	2.32
GO:0030155	regulation of cell adhesion	3.19E-02	2.1
GO:0022409	positive regulation of cell-cell adhesion	3.04E-02	2.2
GO:0045785	positive regulation of cell adhesion	2.88E-02	2.2
GO:1903037	regulation of leukocyte cell-cell adhesion	2.73E-02	2.2
GO:0098542	defense response to other organism	3.23E-02	3.02
GO:0042742	defense response to bacterium	3.09E-02	3.02
GO:0007420	brain development	3.89E-02	15.23
GO:0043207	response to external biotic stimulus	4.34E-02	2.68
GO:1903039	positive regulation of leukocyte cell-cell adhesion	4.25E-02	2.19
GO:0050764	regulation of phagocytosis	4.65E-02	8.8
GO:0050766	positive regulation of phagocytosis	4.48E-02	8.8
GO:0014910	regulation of smooth muscle cell migration	4.43E-02	39.6
GO:0014911	positive regulation of smooth muscle cell migration	4.28E-02	39.6
GO:0022407	regulation of cell-cell adhesion	4.29E-02	2.08
GO:0048878	chemical homeostasis	4.30E-02	10.15
GO:0007165	signal transduction	4.34E-02	1.29
GO:0051707	response to other organism	4.50E-02	2.75
GO:0002478	antigen processing and presentation of exogenous peptide antigen	5.18E-02	1.93

GO:0019884	antigen processing and presentation of exogenous antigen	5.03E-02	1.93
GO:0048522	positive regulation of cellular process	4.99E-02	1.47
GO:0009617	response to bacterium	4.88E-02	2.84
GO:0006956	complement activation	5.36E-02	7.07
GO:0030449	regulation of complement activation	5.23E-02	7.07
GO:2000257	regulation of protein activation cascade	5.10E-02	7.07
GO:0070613	regulation of protein processing	4.97E-02	7.07
GO:0072376	protein activation cascade	4.85E-02	7.07
GO:1903317	regulation of protein maturation	4.74E-02	7.07
GO:0002682	regulation of immune system process	4.95E-02	1.5

Supplementary Table 10. Biological processes gene ontology annotation for gene associated with indel hotspots

GO id	Description	P-value	FDR q-value	Enrichment
GO:0050911	detection of chemical stimulus involved in sensory perception of smell	1.88E-20	2.68E-16	10.11
GO:0050907	detection of chemical stimulus involved in sensory perception	3.11E-19	2.22E-15	9.1
GO:0050906	detection of stimulus involved in sensory perception	5.42E-19	2.58E-15	8.42
GO:0009593	detection of chemical stimulus	2.28E-18	8.14E-15	8.44
GO:0051606	detection of stimulus	5.45E-16	1.56E-12	6.5
GO:0007186	G-protein coupled receptor signaling pathway	6.47E-13	1.54E-09	4.14
GO:0006351	transcription, DNA-templated	1.65E-05	3.36E-02	2.07
GO:0097659	nucleic acid-templated transcription	1.67E-05	2.97E-02	2.07

Supplementary Table 11. Genes associated with rheumatoid arthritis or Juvenile idiopathic arthritis

chr	start	end	gene	known SNPs	novel SNPs	known INDELs	novel INDELs	associated with
1	2487078	2496821	TNFRSF14	21	9	1	0	RA
1	17634690	17690499	PADI4	291	24	19	6	RA
1	38509523	38512450	POU3F1	2	0	1	0	RA
1	114356433	114414381	PTPN22	103	18	16	2	RA
1	117057157	117113661	CD58	72	19	10	2	RA
1	117297007	117311850	CD2	44	8	3	1	RA
1	154377669	154441926	IL6R	183	33	15	3	RA, JIA
1	161475220	161493803	FCGR2A	73	5	4	1	RA
1	161551101	161648444	FCGR2B	381	50	34	8	RA
1	198607801	198726545	PTPRC	372	39	36	22	RA
2	61108656	61158745	REL	80	20	8	4	RA
2	65537985	65659771	SPRED2	404	44	38	13	RA
2	100162323	100759201	AFF3	1606	237	160	45	RA
2	191894302	192016322	STAT4	333	45	27	6	RA, JIA
2	204571198	204603635	CD28	108	13	13	5	RA
2	204732509	204738683	CTLA4	11	0	0	0	RA
2	219246752	219261617	SLC11A1	38	6	5	0	JIA
3	58318607	58411748	PXK	426	28	47	11	RA
4	26165077	26436541	RBPJ	580	103	74	40	RA
4	123372625	123377880	IL2	10	1	0	1	RA
4	123533783	123542224	IL21	24	2	2	0	RA
5	55230923	55290821	IL6ST	116	22	20	9	RA
6	29909037	29913661	HLA-A	153	44	5	18	JIA
6	31321649	31324965	HLA-B	87	32	7	12	RA, JIA
6	31543344	31546113	TNF	4	2	3	1	JIA
6	32546546	32557625	HLA-DRB1	200	68	0	27	RA, JIA
6	32595956	32614839	HLA-DQA1	1084	214	57	107	JIA
6	32627244	32636160	HLA-DQB1	431	251	40	53	JIA
6	33043703	33054978	HLA-DPB1	252	78	29	12	RA, JIA
6	106534195	106557814	PRDM1	38	7	6	2	RA
6	112375275	112392171	WISP3	72	10	1	9	JIA
6	138188351	138204449	TNFAIP3	37	5	5	0	RA, JIA
6	159455500	159466184	TAGAP	43	6	0	0	RA
6	167525295	167553184	CCR6	106	10	4	4	RA
7	22765503	22771621	IL6	23	4	0	1	JIA
7	128577666	128590089	IRF5	44	7	1	0	RA
8	11351510	11422113	BLK	472	51	30	17	RA
9	34709002	34710121	CCL21	2	0	0	0	RA
9	123664671	123691451	TRAF1	123	17	9	2	RA, JIA
9	123714616	123812554	C5	254	39	32	11	RA
10	6052652	6104288	IL2RA	266	20	17	2	RA, JIA
10	6469105	6622263	PRKCQ	577	61	49	15	RA
10	8095567	8117161	GATA3	94	8	10	2	RA
10	63661059	63856703	ARID5B	608	76	43	22	RA
11	36508577	36531822	TRAF6	52	9	9	1	RA
11	60869867	60895324	CD5	59	12	10	2	RA
12	57943781	57980415	KIF5A	65	7	3	2	RA

12	57984957	57997198	PIP4K2C	15	2	2	0	RA
15	38780304	38857776	RASGRP1	212	23	23	8	RA
15	70340129	70390515	TLE3	182	27	13	3	RA
16	85932409	85956215	IRF8	110	16	5	1	RA
17	37921198	38020441	IKZF3	248	32	18	11	RA
19	10461209	10491352	TYK2	93	9	7	1	RA
20	44746911	44758502	CD40	34	6	5	2	RA
21	35885440	35987441	RCAN1	558	35	44	10	RA
21	36160098	37376965	RUNX1	4393	514	395	139	RA
22	24236191	24237414	MIF	8	0	0	0	JIA
22	37521878	37571094	IL2RB	208	19	15	6	RA

RA: rheumatoid arthritis
JIA: juvenile idiopathic arthritis

Supplementary Table 12. JIA samples information

Patient	Gender	Ethnicity	Age of disease onset	ANA status	Sequencing Batch	Achieved Inactive Disease* on Methotrexate (MTX)?
1	F	Caucasian	8	Positive	1	No
2	F	Caucasian	8	Negative	1	Yes
3	F	Caucasian	7	Positive	1	No
4	F	Caucasian	2	Positive	1	Yes
5	F	Caucasian	4	Positive	1	No
6	F	Caucasian	11	Negative	1	Yes
7	F	Caucasian	11	Negative	1	Yes
8	F	Caucasian	12	Negative	1	Yes
9	F	Caucasian	6	Positive	1	No
10	F	Caucasian	8	Negative	1	No
11	F	Caucasian	7	Negative	1	Yes
12	F	Caucasian	4	Positive	1	No.<br>Course complicated by chronic uveitis.
13	F	Caucasian	8	Negative	1	Yes
14	F	Caucasian	15	Negative	1	No
15	F	Caucasian	4	Positive	1	No.<br>Course complicated by chronic uveitis
16	F	Caucasian	10	Negative	1	No
17	F	Caucasian	4	Positive	1	No
18	F	Caucasian	6	Negative	1	Yes
19	F	Caucasian	3	Negative	1	No
20	F	Caucasian	8	Negative	1	No
21	F	Caucasian	10	Negative	1	Yes
22	F	Caucasian	10	Negative	1	No
23	F	Caucasian	4	Positive	1	No.<br>Course complicated by chronic uveitis.
24	F	Caucasian	5	Negative	1	Yes
25	F	Caucasian	3	Positive	1	No
26	F	Caucasian	5	Negative	1	Yes
27	F	Caucasian	12	Negative	1	No
28	F	Caucasian	5	Negative	1	No
29	F	Caucasian	7	Negative	1	No
30	M	Caucasian/ Hispanic	6	Negative	2	No
31	F	Caucasian/ Native American	8	Negative	2	No
32	M	Caucasian/ Hispanic	11	Negative	2	Yes
33	F	Caucasian/ Hispanic	6	Negative	2	No
34	M	Caucasian/ Hispanic	4	Negative	2	No
35	M	Caucasian	9	Negative	2	Yes
36	M	Caucasian	8	Negative	2	Yes
37	M	Caucasian	9	Negative	2	No
38	M	Caucasian	4	Positive	2	No<br>Course complicated by chronic uveitis

39	F	Native American/ Caucasian	12	Negative	2	No
40	F	Caucasian/ Hispanic	3	Positive	2	Follow-up only 8 months. Still had active disease on MTX
41	M	Caucasian	4	Negative	2	Yes
42	M	Caucasian	8	Negative	2	No
43	F	Caucasian/ Native American	9	Negative	2	No
44	F	Native American/ Caucasian	13	Negative	2	Follow-up only 6 months. Still had active disease on MTX
45	M	Native American/ Caucasian	5	Negative	2	Yes
46	M	Caucasian	6	Positive	2	Follow-up only 4 months. Still had active disease on MTX
47	F	Native American/ Caucasian	8	Negative	2	Yes
48	F	Caucasian/ Hispanic	4	Positive	2	Yes

* Inactive disease was determined using the Wallace Criteria: *Arthritis Rheum* 2005; 52: 3354-3562

Supplementary Table 13. Sequencing reads statistics

	Pass QC reads	Properly paired	%properly paired	Mapped	%mapped
Average	843,936,885	812,403,200	97.03	837,863,340	99.27
Maximum	1,068,926,080	1,020,386,014	97.88	1,059,360,024	99.77
Minimum	696,403,720	675,817,666	95.12	693,327,162	97.87
Total	41,352,907,369	39,807,756,780		41,055,303,649	

	Insert sizes	Mean coverage	Number of bases covered	Total bases covered
Average	352	38.74	2,845,046,896	110,985,793,469
Maximum	381	48.61	2,864,577,134	139,266,941,079
Minimum	331	31.55	2,833,724,257	90,373,012,088
Total			136,562,251,006	5,327,318,086,499

Average: average across 48 samples

Supplementary Table 14. Heterozygous to homozygous ratio

	het_known	het novel	het	hom_known	hom_novel	hom	ratio
Average	1,917,071	32,742	1,949,812	1,215,970	927	1,216,897	1.60
Maximum	2,024,913	38,213	2,061,738	1,271,751	1,407	1,272,779	1.70
Minimum	1,794,915	28,606	1,823,521	1,136,401	591	1,137,317	1.49

Het: heterozygous
Hom: homozygous
ratio: het/hom
known: SNPs present in dbSNP141
novel: SNPs not reported in dbSNP141

Supplementary Table 15. Transition to transversion ratio

	Ti_known	Ti_novel	Ti	Tv_known	Tv_novel	Tv	Ti/Tv
Average	2,126,865	21,662	2,148,527	1,006,176	12,007	1,018,183	2.11
Maximum	2,202,969	25,569	2,227,087	1,041,425	13,872	1,055,297	2.118
Minimum	1,999,191	18,945	2,019,521	942,679	10,445	953,521	2.103

Ti: transition
Tv: transversion
known: SNPs present in dbSNP141
novel: SNPs not reported in dbSNP141

Supplementary Table 16. Description of genomic feature and impact for genetic variation

Genomic feature	Description	Impact
DOWNSTREAM	5K bases downstream of a gene	MODIFIER
EXON	Variant hits an exon (from a non-coding transcript) or a retained intron.	MODIFIER
INTERGENIC	Variant is in an intergenic region	MODIFIER
INTRAGENIC	Variant hits a gene, but no transcripts within the gene	MODIFIER
INTRON	Variant hits and intron. Technically, hits no exon in the transcript	MODIFIER
MOTIF	Variant hits TFBSs generates by Jaspar or ENSEMBL	LOW
NEXT_PROT	Variant hits functional sites in a protein	LOW
NON_SYNONYMOUS	Variant causes a codon that produces a different amino acid	MODERATE
SPLICE_SITE	The variant hits a splice acceptor site (defined as two bases before or after exon start, except for the first exon).	HIGH
START_GAINED	A variant in 5'UTR region produces a three base sequence that can be a START codon.	LOW
START_LOST	Variant causes start codon to be mutated into a non-start codon.	HIGH
STOP_GAINED	Variant causes a STOP codon	HIGH
STOP_LOST	Variant causes stop codon to be mutated into a non-stop codon	HIGH
SYNONYMOUS	Variant causes a codon that produces the same amino acid	LOW
UPSTREAM	5K bases upstream of the most distal transcription start site	MODIFIER
UTR_3_PRIME	Variant hits 3'UTR region	MODIFIER
UTR_5_PRIME	Variant hits 5'UTR region	MODIFIER

Supplementary Table 17. Linkage disequibilium (LD) blocks of JIA-associated SNPs

chr	LD start	LD end	rsid	gene
1	25197155	25197586	rs4648881	RUNX3
1	67702526	67705958	rs11209026	IL23R
1	114303808	114377568	rs6679677	PTPN22
1	117688855	117720319	rs2358820	VTCN1
1	154291718	154379369	rs11265608	ATP8B2/IL6R
1	206940310	206947167	rs1800896	IL10
2	100806514	100837567	rs1160542	AFF3
2	100813331	100837567	rs6740838	AFF3/LONRF2
2	113488188	113521754	rs6712572	IL1
2	113528872	113537352	rs2071374	IL1
2	191874247	191874247	rs45539732#	STAT1
2	191875901	191892308	rs13029532#	STAT1
2	191900449	191935804	rs3821236	STAT4
2	191943742	191973034	rs10174238	STAT4
2	216190654	216205267	rs12995526	ATIC
3	46183180	46323679	rs79893749	CCR1/CCR3
3	119125202	119247898	rs4688013	TIMMDC1/CD80
4	123141054	123548068	rs17388568	IL2-IL21
4	123309902	123540758	rs1479924	IL2/IL21
4	123509421	123551114	rs6822844	IL2-IL21
5	55440730	55442249	rs71624119	ANKRD55
5	96121715	96126308	rs30187	ERAP1
5	96220087	96373750	rs27290	ERAP2/LNPEP
5	131813219	131832514	rs4705862	c5orf56/IRF1
5	159911506	159914665	rs2910164	IL12A
6	31492453	31543031	rs1800629	TNFA/TNF
6	112359543	112448654	rs2280153	WISP3
6	137959235	138006504	rs6920220	TNFAIP3
6	137961013	138011151	rs13207033	TNFAIP3
7	22766221	22768124	rs1800795	IL6
7	22774437	22811384	rs7808122	IL6
7	28152193	28243473	rs10280937	JAZF1
7	87138511	87139316	rs1045642	ABCB1
7	92239144	92250140	rs42041	CDK6
7	128576023	128578301	rs2004640	IRF5
8	63947643	63977454	rs1800909	GGH
8	108192501	108270428	rs1010824	ANGPT1
9	123636121	123723351	rs10818488	TRAF1-C5
10	6078553	6097283	rs7909519	IL2RA
10	72360387	72378489	rs35947132	PRF1
10	90759613	90764891	rs7069750	FAS
11	36336263	36371757	rs4755450	PRR5L
12	6495275	6511996	rs2364480	LTBR
12	60113674	60242004	rs10877333	SLC16A7
12	109898913	110033734	rs11836136	MVK
12	109918103	110047595	rs7957619	MVK
12	111884608	112007756	rs3184504	SH2B3/ATXN2
13	40299842	40368601	rs7993214	COG6

13	43056036	43066523	rs34132030	Chr13q14
14	69250891	69260588	rs12434551	ZFP36L1
16	11400900	11435990	rs66718203	PRM1/RMI2
17	48277749	48278507	rs1800012	COL1A1
18	12774326	12809340	rs2847293	PTPN2
18	12821903	12880206	rs7234029	PTPN2
18	12857758	12885120	rs149850873#	PTPN2
18	67513805	67543688	rs763361	CD226
19	10427721	10463118	rs34536443	TYK2
19	41858876	41875573	rs1800471	TGFB1
21	36712588	36715761	rs9979383	RUNX1
21	46932383	46959087	rs1051266	SLC19A1
22	21916166	21983260	rs2266959	UBE2L3
22	24234493	24237862	rs755622	MIF
22	37531436	37537058	rs2284033	IL2RB

Supplementary Table 18. Dataset of epigenetic elements that are used for integration with JIA genetic variants

**Cell type**<br>Epigenetic elements	GEO accession/sample ID
**CD4+ T cells**	
H3K27ac	GSM1220560
H3K27me3	*MS010308
H3K4me1	GSM1220567
H3K4me3	GSM1102798
DNase HS	GSM1008572
CTCF	GSM325895
**CD20+ B cells**	
H3K27ac	GSM1003459
H3K27me3	GSM1129357
H3K4me1	GSM733772
H3K4me3	GSM945229
DNase HS	GSM1008588
CTCF	GSM1003474
**CD14+ Monocytes**	
H3K27ac	GSM1003559
H3K27me3	GSM1003564
H3K4me1	GSM1003535
H3K4me3	GSM1003536
DNase HS	GSM1008582
CTCF	GSM1003508

DNase HS: DNase hypersensitivity
* International Human Epigenome Consortium Data, McGill Epigenomics Mapping Centre (2015). Dataset from EGA Study EGAS00001000995. Available from http://epigenomesportal.ca/edcc.
